# An adaptive biodegradable zinc alloy with bidirectional regulation of bone homeostasis for treating fractures and aged bone defects

**DOI:** 10.1016/j.bioactmat.2024.04.027

**Published:** 2024-05-06

**Authors:** Jialian Xu, Guo Bao, Bo Jia, Minqi Wang, Peng Wen, Tianyou Kan, Shutao Zhang, Aobo Liu, Haozheng Tang, Hongtao Yang, Bing Yue, Kerong Dai, Yufeng Zheng, Xinhua Qu

**Affiliations:** a Department of Bone and Joint Surgery, Department of Orthopedics, Renji Hospital, Shanghai Jiaotong University School of Medicine, Shanghai, 200001, China; b Laboratory Animal centre, National Research Institute for Family Planning, Beijing, 100081, China; c Department of Mechanical Engineering, Tsinghua University, Beijing, 100084, China; d School of Engineering Medicine, Beihang University, Beijing, 100191, China; e Department of Orthopedics, Shanghai Key Laboratory of Orthopedic Implant, Shanghai Ninth People's Hospital, Shanghai Jiao Tong University School of Medicine, Shanghai, 200011, China; f School of Materials Science and Engineering, Peking University, Beijing, 100871, China

**Keywords:** Aberrant bone metabolism, Bone healing, Biodegradable materials, Zn-0.8 Mg alloy, Bidirectional regulation

## Abstract

Healing of fractures or bone defects is significantly hindered by overactivated osteoclasts and inhibited osteogenesis in patients with abnormal bone metabolism. Current clinical approaches using titanium alloys or stainless steel provide mechanical support but have no biological effects on bone regeneration. Therefore, designing and fabricating degradable metal materials with sufficient mechanical strength and bidirectional regulation of both osteoblasts and osteoclasts is a substantial challenge. Here, this study first reported an adaptive biodegradable Zn-0.8 Mg alloy with bidirectional regulation of bone homeostasis, which promotes osteogenic differentiation by activating the Pi3k/Akt pathway and inhibits osteoclast differentiation by inhibiting the GRB2/ERK pathway. The *anti*-osteolytic ability of the Zn-0.8 Mg alloy was verified in a mouse calvarial osteolysis model and its suitability for internal fracture fixation with high-strength screws was confirmed in the rabbit femoral condyle fracture model. Furthermore, in an aged postmenopausal rat femoral condyle defect model, 3D printed Zn-0.8 Mg scaffolds promoted excellent bone regeneration through adaptive structures with good mechanical properties and bidirectionally regulated bone metabolism, enabling personalized bone defect repair. These findings demonstrate the substantial potential of the Zn-0.8 Mg alloy for treating fractures or bone defects in patients with aberrant bone metabolism.

## Highlights

• The biodegradable Zn-0.8 Mg alloy was investigated for bone regeneration of different orthopedic applications.
• Zn-0.8 Mg alloy outstanding regulated bone homeostasis by suppressing osteoclast differentiation and promoting osteogenesis.
• Zn-0.8 Mg alloy significantly inhibited the Ti particle-induced calvarial osteolysis in mouse model.
• A rabbit femoral condyle fracture model indicated the great internal fracture fixation properties of Zn-0.8 Mg alloy.
• 3D printed Zn-0.8 Mg alloy scaffold exhibited outstanding bone healing performance for aged rat femoral condyle defect model.

## Graphical abstract

**Figure:**
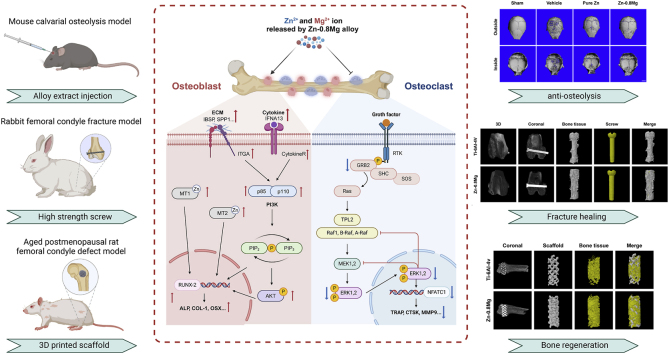

## 1 Introduction

Fractures and bone defects are common clinical orthopedic conditions. Especially among our aging population, the rate of fractures and bone defects continues to rise, with new fractures increasing by 33.4 % since 1990, resulting in approximately 162–196 million new annual cases worldwide [1]. Additionally, 2 million bone defect surgeries are performed annually worldwide [2]. Factors causing aberrant bone metabolism, including co-infections, severe trauma, advanced age, postmenopausal osteoporosis, bone metastases, use of certain medications (e.g., glucocorticoids, diuretics, etc.), autoimmune disease (e.g., rheumatoid arthritis), renal insufficiency, hyperparathyroidism function, hereditary bone disease, and diabetes mellitus affect the bone conversion process through varied and complex pathways [[3], [4], [5]]. However, they all lead to increased osteoclast bone resorption and decreased osteoblast bone formation, resulting in a metabolic imbalance in the systemic or local skeletal microenvironment [6]. A key factor in bone healing is the dynamic balance of bone metabolism. Therefore, patients with aberrant bone metabolism are more prone to delayed or even a lack of healing of fractures or defects than healthy patients, which impacts their quality of life as well as the economy [7,8].

Traditional metals, like titanium and stainless steel, offer good mechanical qualities and biological inertness, making them frequently employed in clinical bone repair and internal fixation surgery [9]. However, in patients with bone fractures or defects combined with aberrant bone metabolism, inert metallic materials lack the biological effect of improving the bone regeneration microenvironment [10]. In the bone microenvironment, bone resorption is more pronounced than bone formation, which leads to early limitations in bone integration on the implant surface, resulting in aseptic loosening and other risks [11]. Surface modification and the use of anti-osteoporosis drugs are still the primary methods for addressing the weak biological effects of inert metals; however, these methods are hindered by complex manufacturing processes, high costs, and unstable drug release [12]. Thus, developing fundamental implantable materials with improved bone metabolism that can efficiently promote bone healing in patients with aberrant bone metabolism represents a more practical solution for clinical applications.

Biodegradable metallic materials have shown promise in treating fractures and bone defects for the past several years [13]. These materials good mechanical fitness and biosafety and gradually corrode and degrade in the human environment, providing space for regenerating bone tissues, releasing safe concentrations of metal ions, and performing certain biological functions. Zn-based alloys have attracted considerable attention as a next-generation of biodegradable metals. Compared to magnesium (Mg)-based alloys, which demonstrate a rapid and uncontrollable degradation rate, irregular pitting corrosion, and gas production, zinc-based alloys boast an acceptable degradation rate, harmless degradation products, and good biocompatibility, exhibiting better prospects in the field of bone implant applications [14]. Zinc is not only an essential element for physiological activities, including enzymes in the human body, but also a second messenger for transmitting signals such as calcium. Zinc can inhibit osteoclast differentiation, promote osteogenic differentiation, activity, cell proliferation, angiogenesis, and immunomodulatory functions in a dose-dependent manner [15]. Consequently, Zn-based alloys are expected to increase the healing rate of patients with refractory fractures/bone defects by modulating bone metabolic processes.

In our previous research, we extensively developed and evaluated biodegradable zinc-based alloys. Our findings indicate that binary zinc alloys, when prepared with various alloying elements, exhibit distinct characteristics. These characteristics allow for the design of clinical application scenarios and modalities that are tailored to their specific functions. For example, Zn–Li alloys exhibit outstanding mechanical properties and are appropriate for use as plate and screw materials for strong internal fixation procedures in high-load-bearing areas [16]. Moreover, the antimicrobial characteristics of Zn–Ag and Zn–Cu alloys have great potential for infection-related orthopedic surgery [17,18]. In addition, Zn–Sr alloys can promote osteogenic differentiation and angiogenesis and is an innovative osteoinductive material for addressing bone abnormalities in load-bearing areas [19]. However, for the complex situation of fractures/bone defects with aberrant bone metabolism, the ideal biological effect is specific bidirectional regulation, that is, the inhibition of osteoclasts and induction of osteogenesis to restore the bone-remodeling microenvironment and promote bone healing [20]. Although osteoinductivity is a commonly discussed aspect of orthopedic implants, osteoclast inhibition has not been thoroughly investigated. Mg, as an essential element for bone growth and metabolism, was the first and most common alloy added to a zinc-based metal to optimize the mechanical characteristics and biocompatibility of pure Zn [21]. The addition of Mg^2+^ can help promote osteogenesis by regulating the Pi3k/Akt pathways [22]. Moreover, Mg^2+^ serves as an effective antagonist of Ca^2+^ channels, whereas Ca^2+^ is closely associated with osteoclast differentiation and bone resorption [23]. Therefore, Zn–Mg alloys represent novel and feasible therapeutic implant materials for fractures/bone defects in patients with aberrant bone metabolism. However, studies of the dual regulation mechanism of bone metabolism by Zn–Mg alloys, or their function in multiple animal models, are lacking. In our previous comprehensive evaluation of Zn binary alloys prepared with eight different alloying elements (including Mg) and different mass ratios, Zn-0.8 Mg alloys demonstrated ideal performance for bone repair implants with respect to the mechanical properties, cytocompatibility, osteogenesis, and osseointegration [24].

Subsequently, in this study, we conduct a thorough analysis of the Zn-0.8 Mg alloy to assess its potential as a therapeutic material for fractures and bone defects in patients with bone metabolism disorders. Specifically, we use three distinct animal models (osteolysis, internal fixation of fractures, and bone defects in senescent postmenopausal rats) to simulate clinical scenarios that address the following questions: (1) How do osteoblasts, osteoclasts, and neutrophils respond biologically to the Zn-0.8 Mg alloy, and what are the underlying mechanisms? (2) Can the Zn-0.8 Mg alloy significantly inhibit bone resorption in osteoclast-activated osteolysis models? (3) How effective are Zn-0.8 Mg alloy screws in treating femoral condyle fractures compared to Ti–6Al–4V screws? (4) Can Zn-0.8 Mg alloy scaffolds prepared using 3D printing accurately treat bone defects in the context of aberrant bone metabolism in aged postmenopausal femurs?

## 2 Methods

### 2.1 Preparation of Zn–Mg alloy

Zn-0.8 Mg alloys were produced by blending pure zinc (99.99 %) and magnesium powders (99.9 %). They were alloyed according to Mg mass percentages of 0 and 0.8 %, respectively. The metal blocks underwent thorough homogenization at 350 °C for 48 h and were subsequently quenched with water prior to being extruded. Subsequently, the metal ingots underwent extrusion at 260 °C, maintaining a 36:1 ratio. The metal rods were cut into disks (Φ 10 × 1 mm) and cylinders (Φ1.5 × 20 mm), then mechanically polished to 2000 mesh. According to our previous work, the porous scaffolds (Φ 3 × 4 mm) of Ti alloy and Zn–Mg alloy were prepared with laser powder bed fusion (L-PBF) process [25].

The metal samples were cleaned in ethanol and acetone via sonication and dried at room temperature. Before conducting the cytotoxicity and animal experiments, the metal samples were sterilized using ethylene oxide. The extraction solution was prepared according to the ISO 10993 standard. We immersed the sterilized pure Zn and Zn–Mg metal disks in the α-MEM medium at a ratio of 1.25 mL/cm^2^ and then placed them in an incubator (37 °C, 5 % CO_2_). The extraction solution was filtered using sterile syringe filters.

### 2.2 Zn–Mg alloy microstructure characteristics

The samples were polished to 7000 grit before observation. The metallographic structures of the alloys were examined with optical microscopy. A diffractometer (XRD; scanning process settings: speed 2°/min, range 10°–90°) with Cu Kα radiation (λ = 1.5406 Å) was applied to analyze microstructures and composition of the alloy materials. Analyzed the microstructures and compositions of alloy materials.

### 2.3 Mechanical properties evaluation

The cylinder and scaffold samples used in the mechanical testing were processed according to the ASTM specifications. The mechanical properties were evaluated using a universal material testing machine (Instron). The displacement rates for tensile and compressive tests were 1 × 10^−4^ s^−1^ and 2 × 10^−4^ s^−1^, respectively. The ultimate compressive strength was defined as the maximum stress reached before 50 % of the compressive stress.

### 2.4 Electrochemical and immersion tests

Electrochemical tests were implemented at room temperature in simulated body fluid (SBF) solution. A three-electrode system, including a saturated calomel electrode (SCE) and a platinum counter electrode, was selected for the electrochemical tests. Each sample was monitored for its open-circuit potential (OCP) for 3600 s. Electrochemical impedance spectroscopy (EIS) was performed in the frequency range of 10^5^ Hz to 10^−2^ Hz by applying a perturbation of 10 mV. The corrosion potential of the sample surface in the SBF solution for 24 h was measured using a scanning vibrating electrode technique (SVET) system (Applicable Electronics Inc.).

### 2.5 In vitro cell culture

Mouse bone marrow-derived macrophages (BMMs) and the mononuclear macrophage cell line Raw 264.7 cells were selected as representative cells to investigate the cytocompatibility and osteoclast differentiation of the metal materials. The mouse osteoblastic cell line MC3T3-E1 was selected as a representative cell line to evaluate the osteogenic differentiation of the metal materials. The MC3T3-E1 cells and BMMs were cultured in α-MEM culture medium (Gibco), which supplemented with 10 % FBS and 1 % penicillin/streptomycin (P/S, Gibco) at 37 °C with 5 % CO2.

### 2.6 Cytocompatibility of metal materials

The cytocompatibilities were evaluated with Cell Counting Kit-8 (Dojindo). The BMMs were collected and manually counted, and the cell concentration was adjusted to 2 × 10^4^ cells/mL. The suspended cells were added to a 96-well plate (100 μl/well) and pre-incubated in a humidified incubator (at 37 °C, 5 % CO_2_) for 24 h. Then, the culture medium of the treatment group was replaced with the extraction solution of metal materials. The blank control group was replaced by the α-MEM culture medium. After 24, 48, and 72 h of co-culture, according to the technical manual, we added 10 μL of CCK-8 solution to each well and incubated for 1 h. The absorbance of each well was measured at 450 nm using a microplate reader (BioTek, Synergy H1, USA). Each group underwent a minimum of five measurements on average.

### 2.7 ALP staining to evaluate osteoblast differentiation

The MC3T3-E1 cells were collected and manually counted, and the cell concentration was adjusted to 2 × 10^4^ cells/mL. The suspended cells were added into a 48-well plate (250 μl/well) with α-MEM culture medium and pre-incubated in a humidified incubator (37 °C, 5 % CO_2_). Subsequently, upon reaching sub-confluence, switched to osteogenic induction medium containing either basic α-MEM medium or the extract solution of metal materials, to promote osteoblast differentiation. Every two days, the culture medium underwent renewal. After 7 and 14 days, an ALP staining kit (Wako) was applied to evaluate osteoblast differentiation of MC3T3-E1 cells.

### 2.8 TRAP staining to evaluate osteoclast differentiation

The suspended cells were added into a 24-well plate (500 μl/well) with α-MEM culture medium containing M-CSF (30 ng/mL) and pre-incubated in a humidified incubator (at 37 °C, 5 % CO_2_) overnight. The culture medium was replaced with an extract solution of metallic materials (containing 30 ng/mL M-CSF and 50 ng/mL RANKL) to induce osteoclasts. Every two days, the culture medium underwent renewal. After observing multinuclear giant cells (MNGCs) using light microscopy (5–7 days of culture), a TRAP staining kit (Wako) was used to identify osteoclasts.

### 2.9 Osteoclast bone resorption

The BMMs were collected and manually counted, and the cell concentration was adjusted to 8 × 10^4^ cells/mL. The sterilized calf bone slices were added into each well of a 96-well plate; subsequently, suspended cells were added into a 96-well plate (100 μl/well) with α-MEM culture medium containing M-CSF (30 ng/mL) and pre-incubated. ng/mL) and pre-incubated the plate in a humidified incubator (at 37 °C, 5 % CO_2_) overnight. The culture medium was replaced with α-MEM containing M-CSF (30 ng/mL) and RANKL (50 ng/mL) for four consecutive days. The culture medium was then adding the extraction solution of metallic materials. The culture medium containing M-CSF, RANKL and extraction solution was changed every other day. Bone slices were collected, rinsed with sterile PBS, fixed with 2.5 % glutaric dialdehyde. The samples were then subjected to dehydration using different concentration of ethanol concentrations. The absorption of osseous lacunae in the bone slices was observed using an SEM.

### 2.10 Quantitative real-time PCR

The mRNA expression levels in MC3T3-E1 cells, BMMs and neutrophils were determined by real-time PCR. The total RNA was extracted using a RNeasy micro kit (Qiagen) and subsequently reverse-transcribed to cDNA using a QuantiTect reverse-transcription kit, in according to the manufacturer's instructions. Quantitative real-time PCR was performed using 2 × TB Green Premix Ex Tavern II (Takara) on a QuantStudio 7 Flex Real-Time PCR System (Thermo Fisher Scientific). The determination of cycle threshold values for each gene was followed by normalization to the internal reference gene β-actin, or GAPDH. The primers of interest are shown in Tables S1, 2, 3.

### 2.11 Proteomics and Parallel reaction monitoring (PRM) quantitative assay

TMT quantitative proteomics was used to analyze the protein components of RAW cells. with α-MEM medium containing M-CSF (30 ng/mL) for 24 h. The plates were placed in a humidified incubator at 37 °C with 5 % CO_2_. The culture medium was then replaced with the extract solution of Zn-0.8 Mg (containing 50 ng/mL RANKL and 30 ng/mL M-CSF) for osteoclast induction. Every two days, the culture medium underwent a renewal process. After four to five days of culture in the induction medium, typical osteoclast morphology was observed by optical microscopy. Cells were gathered following a triple rinse with PBS. The cells were lysed in RIPA lysis buffer containing 1 mM phenylmethanesulfonyl chloride. After centrifugation, carefully collected the liquid supernatant and quantified the protein concentration using a BCA Protein Assay Kit (Thermo Fisher Scientific). Sodium dodecyl sulfate-polyacrylamide gel electrophoresis was utilized to ascertain the proteins' purity and molecular mass. Trypsin was employed to enzymatize the proteins, which were then marked with TMT. Equal amounts of each labeled sample were mixed into one sample and chromatographically separated. The final samples were analyzed by LC-MS/MS (Thermofisher Scientific, USA). Proteomic data were recorded and analyzed with reference to a bioinformatics database. Proteins of interest were selected, and PRM was used to verify the expression of interest protein.

### 2.12 Activation of neutrophil *in vitro*

Neutrophils were separated from C57BL/6 mice using density-gradient centrifugation. Briefly, Bone marrow (BM) cells from mouse femurs were rinsed with PBS containing 2 mM EDTA and filtered through a 70 μm cell strainer. BM cells were centrifuged with 62 % Percoll to obtain purified neutrophils. After lysing with red blood cell lysis buffer, the purity of neutrophils from the final cells was 90 ± 2 %. Lipopolysaccharide (LPS) was selected as the inflammatory stimulus. The neutrophils were treated with RPMI 1640 culture medium supplemented with 100 ng/mL LPS for 4 and 8 h. QPCR was applied to investigate the activation status of neutrophils.

### 2.13 RNA-sequencing

The mRNA expression was detected by RNA sequencing (Shanghai Xu Ran Biotechnology Co., Ltd.). Using STAR, clean reads were matched to the mouse genome (mm10) from the collection, permitting a single discrepancy. StringTie (v1.3.1c) was used to generate the gene expression data, and differential gene expression was analyzed using DESeq2 (v1.16.1). Criteria for identifying different expressed genes (DEGs) included a P-value less than 0.05 and an absolute fold change of 2 or more. Subsequently, DEGs were chosen for analyses focusing on functional and signaling pathway enrichment, utilizing the TopGO and KEGG databases.

### 2.14 In vivo surgical procedure of animal experiment

All animal experiment adhered to the animal life protection standards and procedures sanctioned by Shanghai Rat&Mouse Biotech Co., Ltd (Issue No. 20230213 (14)).

The mouse cranial osteolysis model was established by calvaria subperiosteal injecting 0.1 mL of Ti particle suspension (suspend with sterile PBS to 300 ng/mL). Forty male C57BL/6 mice, each eight weeks old, were randomly assigned into four distinct groups: sham, vehicle, Pure Zn, and Zn-0.8 Mg. A daily injection of either extract solution of metal materials or PBS was administered into the subperiosteum. Bone samples were gathered post 14 days for further examination.

The femoral condyle fracture was established in 20 rabbits (three-month-old, body weight = 2.2–2.5 kg). Briefly, the femoral condyle was exposed using a lateral parapatellar approach. The lateral condyle of the femur was subsequently subjected to longitudinal fractures using a pendulum saw with a thickness of 1 mm and then stabilized using Ti–6Al–4V or Zn-0.8 Mg alloy screws, which were positioned perpendicular to the fracture line. The bone tissues were collected after 8 months post-surgery for subsequent analysis.

A model for repairing cylindrical bone defects in the lateral femoral condyle was established in 20 twenty-four-month-old female Sprague-Dawley rats. Briefly, a cylindroid bone defect (Φ3 mm × 4 mm) was created in femoral lateral condyle using a drill bit. After removing the fragmented bone within drill pit, the Ti–6Al–4V or Zn-0.8 Mg alloy scaffold was implanted into the defect. The scaffold was implanted following bone rinsing with normal saline solution. Subsequent analysis was conducted three months post-surgery on the collected bone tissues.

### 2.15 Imaging evaluation

Radiographs of the rabbit fractures were obtained on the day of the surgical procedure and three months after the surgical procedure to evaluate the progress of the bone tissues of the experimental animals, which were collected without any damage and then fixed in 4 % paraformaldehyde. Subsequently, the samples were subjected to micro-CT scanning. The region of interest (ROI) was defined as the 1 mm (rat and rabbit model) or 3 mm (mouse model) peripheral area surrounding the operating position. Various bone-related parameters were measured within this ROI.

### 2.16 Histomorphometric evaluation

The fixed specimens were dehydrated using an ethanol gradient, made transparent with xylene, and embedded in methyl methacrylate. The slice thickness is ground to less than 100 μm and stained with either Methylene Blue Acid Fuchsin (MB-BF), Paragon, or Van Giseon.

After a 4-week decalcification process in 10 % EDTA, the tissue sections were meticulously extracted. These specimens were subsequently dehydrated, made transparent, paraffin embedded, sliced, and stained. Immunohistochemical assay was conducted to assess osteoclast-related proteins, osteogenic proteins, as well as common inflammatory markers.

### 2.17 Statistical analysis

Imaging analysis was performed using the ImageJ software. The statistical significance of the data was evaluated using Student's t-test, one-way analysis of variance (ANOVA) followed by Tukey's post-hoc testing with GraphPad Prism 9.0. All values were presented as mean ± SD (n ≥ 3) and were considered significant at P < 0.05. (*P < 0.05, **P < 0.01, ***P < 0.001).

## 3 Results and discussion

### 3.1 Material characterization and biocompatibility

**Fig. 1:**
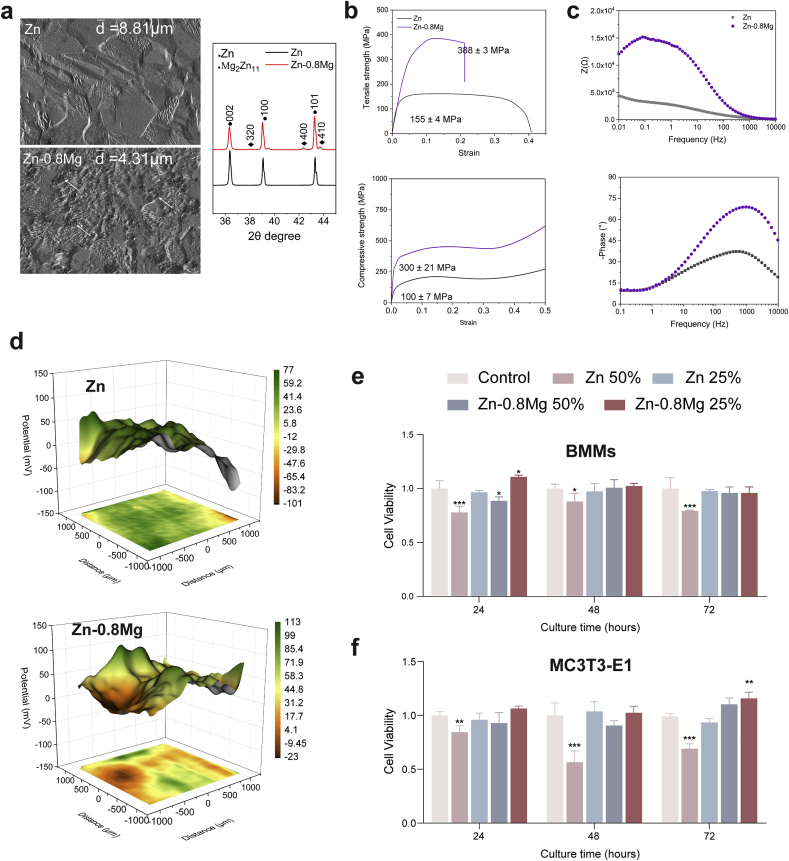
Material characterization and biocompatibility. **a**) Metallographic images and X-ray diffraction patterns. Intermetallic phase is marked with a white arrow. **b**) Tensile and compressive curves; values in tensile and compressive tests are the ultimate tensile strength and compressive yield strength, respectively (n = 3). **c**) Bode plots of samples in SBF solution. **d**) Scanning vibrating electrode monitoring of surface potential on the sample in SBF solution. **e**) and **f**) Cell viability of pure Zn and Zn-0.8 Mg alloy. (n = 6).

The performance of Zn-0.8 Mg alloys was assessed in respect of microstructure, mechanical performance, and electrochemical behavior. Pure Zn mainly contained equiaxed grains of approximately 8.81 μm; the addition of Mg (0.8 wt%) drastically reduced the grain size of Zn–Mg alloys to 4.31 μm, which were characterized by dendritic α-Zn and Zn + Mg_2_Zn_11_ intermetallic compounds distributed at the junctions. The Mg_2_Zn_11_ phase was present in X-ray diffraction patterns and was evenly distributed in the Zn matrix according to the metallographic image (Fig. 1a). Magnesium has a low solubility limit in the mechanical alloying process with zinc. Different production techniques have a significant impact on the mechanical characteristics of metallic materials [26,27]. After restricting the preparation process as extrusion, Zn-0.8 Mg exhibits the best superior compressive and tensile properties in the Zn-based alloy with 0-0.8 wt % Mg, albeit with low elongation [24]. Compared to pure Zn, the ultimate tensile strength of the Zn-0.8 Mg alloy was doubled and the elongation to failure rate was halved. Moreover, the compressive yield strength of the Zn-0.8 Mg alloy was three times that of pure Zn (Fig. 1b). Compared with other binary degradable zinc alloys (including Zn–Cu, Zn–Li, Zn–Sr, Zn–Fe, Zn–Ag, Zn–Mn, Zn–Ca), the tensile strength and compressive strength of the Zn-0.8 Mg alloy were second only to those of the Zn–Li alloy [24]. The Zn–Mg alloy not only met the tensile strength requirement of >300 MPa for implant applications, but also exhibited high plasticity, which can be adapted to the requirements of various orthopedic implants, such as high-strength bone screws and porous scaffolders [27]. Moreover, the tensile and compressive strength of Zn-0.8 Mg were close to those (115.06 ± 16.36 MPa and 210 ± 80 MPa, respectively) of human femoral cortical bone tissue [28,29]; thus, the Zn-0.8 Mg alloy can provide a good biomechanical fit and mechanical support at the load-bearing bone site.

Upon contact with SBF solution, the surface potential of the Zn-0.8 Mg alloy was clearly enhanced from that of pure Zn because of the existence of two phases (Fig. 1d). However, the addition of Mg also increased the surface impedance at medium and low frequencies, indicating greater protection of corrosion products (Fig. 1c). The degradation processes of the pure Zn and Zn–Mg alloy are comparable in that released Zn^2+^ reacts with OH- to form degradation products such as Zn oxides and hydroxides [30,31]. The latter can further form soluble chlorinated salts in high chloride ion environments, so does not theoretically produce a strongly alkaline microenvironment upon gas release. Thus, our results are consistent with previous results in that galvanic coupling corrosion occurred in the SBF solution of Zn-0.8 Mg because of the potential difference between the two metals, which increased the degradation rate of the Zn-0.8 Mg alloy in contrast to pure Zn.

In order to evaluate the clinical potential of Zn-0.8 Mg, the laser powder bed fusion process was used to prepare Zn-0.8 Mg scaffolds with different porosities (20, 50, and 80 %). The microstructure of Zn-0.8 Mg scaffolds was characterized using SEM (Fig. S1a). No visible cracks or pores were observed inside the scaffold, indicating that construction was sound, and the degree of densification was high. The mechanical properties of the scaffolds were also determined. As the porosity increased, the compressive strength and modulus decreased continuously (Figs. S1b and c). As shown in Fig. S2, the average grain size of printed Zn-0.8 Mg alloy is 2.25 μm compared to 4.31 μm of as-extruded Zn-0.8 Mg alloy. The rapid solidification of laser melting further reduces the grain size of Zn–Mg alloys. Additionally, the as-extruded sample consists mainly of equiaxed grain while the grain growth along the solidification direction in the printed sample.

In addition, the recommendatory quotidian intake of Zn (6.5–15 mg) is much lower compared to the common biodegradable metal Mg (375–700 mg), with organisms exhibiting more sensitive concentration-dependent effects of Zn^2+^ [32,33]. Therefore, different concentrations of metal extracts (25 %, 50 %) were prepared, and the potential effects of Zn-0.8 Mg alloy on local bone metabolism cells (osteogenic differentiation/osteoclastic activation) were evaluated *in vitro*. Following the one-fold dilution, the pure Zn group showed significant cytotoxicity in both two cells at three time points. The Zn-0.8 Mg alloy group exhibited cytotoxicity to BMMs only at the 24-h time point and no significant cytotoxicity to MC3T3-E1 cells. However, after two-fold dilution, both extracts showed better cellular biosafety against MC3T3-E1 cells and BMMs (Fig. 1e and f). Surprisingly, the 25 % Zn-0.8 Mg alloy extract was even able to promote the proliferation of both cells. *In vitro* cytocompatibility tests showed that alloying with Mg markedly improved the cytocompatibility of Zn. To exclude interference from the cytotoxicity of the extract, subsequent *in vitro* experiments were performed using twice-diluted extract.

### 3.2 Influence and mechanism of Zn-0.8 Mg alloy on osteogenic differentiation *in vitro*

**Fig. 2:**
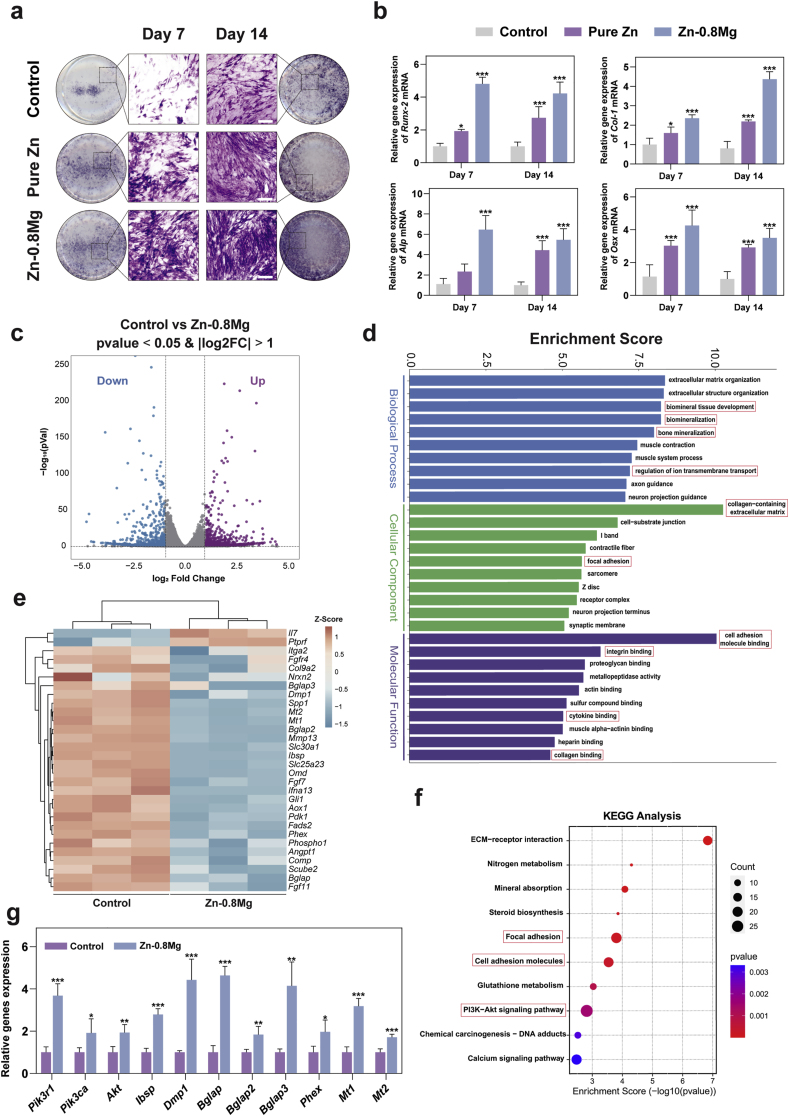
Influence of Zn-0.8 Mg alloy extract on osteogenic differentiation. **a**) Representative alkaline phosphatase (ALP) staining of MC3T3-E1 cells (culture for 7 and 14 days). Scale bar: 100 μm. **b**) mRNA expression of osteogenic genes (*Runx-2, Alp, Col-1,* and *Osx*) in MC3T3-E1 cells after 7 and 14 days treated with metal extract (n = 4). **c**) and **e**) Volcano map and heat map showing the differential mRNA expression of MC3T3-E1 cells (culture for 14 days). **d**) and **f**) Enrichment analysis results of GO and KEGG pathways. Red box highlights important pathways that may be involved in osteogenic differentiation regulation by Zn-0.8 Mg alloy. **g**) qPCR was used to verify the changes of key genes involved in regulating osteogenic differentiation obtained by RNA-seq analysis (n = 4).

Osteoblasts are essential for bone metabolism and give important play to bone healing. The influence of the Zn-0.8 Mg alloy on osteoblast differentiation was assessed by adding metal extraction solutions into osteogenic differentiation culture medium to stimulate the osteogenic differentiation of MC3T3-E1 cells. Osteogenic activity was assessed by ALP staining. After 7 and 14 days of co-cultivation, deeper ALP staining was observed after 14 days and in the Zn-0.8 Mg group (Fig. 2a). Additionally, genes related to osteogenic differentiation (*Runx-2, Alp, Col-1,* and *Osx*) were evaluated using qPCR; the resulting expression of osteogenic differentiation genes in two groups was in line with the ALP staining results (Fig. 2b). Overall, *in vitro* studies demonstrated excellent osteogenesis promotion with both two groups. The addition of Zn-0.8 Mg extracts further enhanced the expression of osteogenesis-related genes compared with pure Zn.

Therefore, we explored the synergistic osteogenic mechanism of Zn-0.8 Mg alloy degradation products. RNA-seq analysis was conducted on MC3T3-E1 cells after 7 and 14 days of osteogenic induction and control groups to elucidate the mechanism by which the Zn-0.8 Mg alloy promotes osteogenic differentiation. After 7 days of induction, the Zn-0.8 Mg alloy group indicated 1478 differential genes in contrast to the control group, with 593 upregulated and 885 downregulated genes (P < 0.05, |log 2 FoldChange| ≥ 1), as shown in Fig. S3a. Notably, osteogenesis-related genes (*Spp1*, *Bglap2*, *Bglap3*) and metallothionein 1/2 (*Mt1*, *Mt2*) showed significant upregulation (Fig. S3b). GO and KEGG analysis highlighted enrichment in pathways such as extracellular matrix (ECM)-receptor interactions (Figs. S3c and d).

**Fig. 3:**
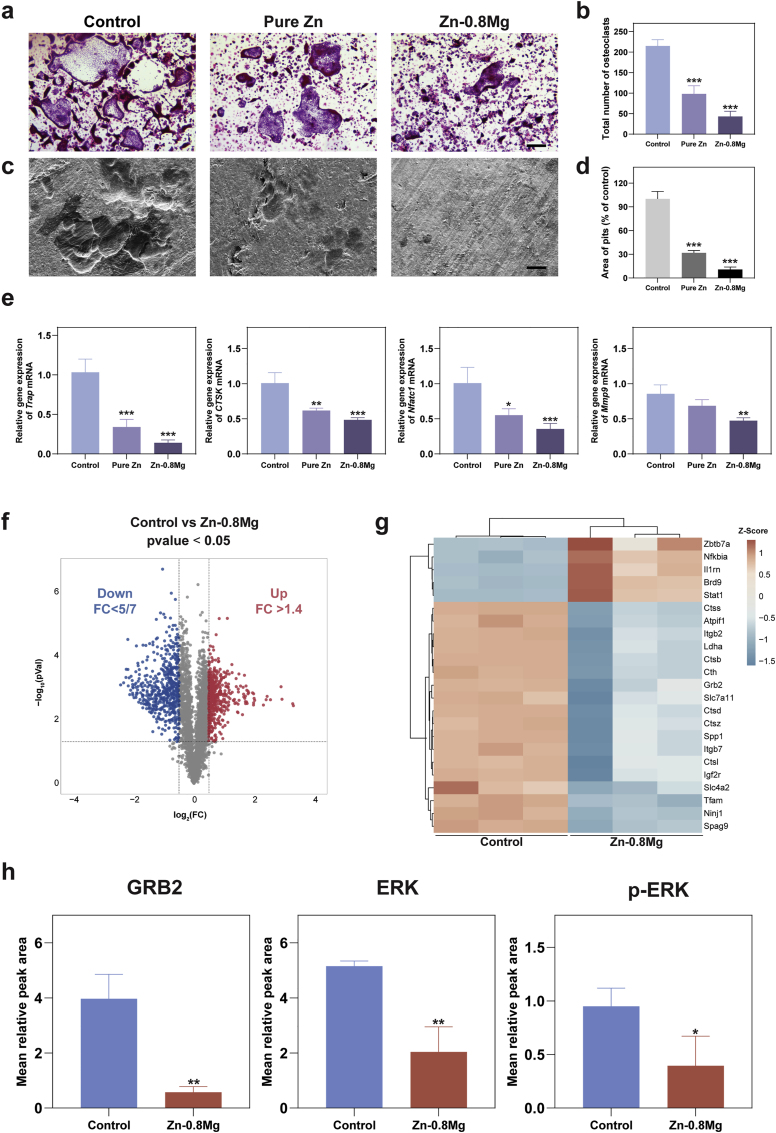
Influence of Zn-0.8 Mg alloy extract on osteoclast differentiation. **a**) Representative TRAP staining of BMMs cultured with metal extract and osteoclast differentiation stimulation for five days. Scale bar: 100 μm. **b**) Quantitative analysis of the number of TRAP-positive osteoclasts in (**a**) (n = 3). **c**) Scanning electron microscopy images of bone resorption pits. Scale bar: 25 μm; **d**) Quantitative analysis of the resorption pit areas in (**c**) (n = 3). **e**) mRNA expression of osteoclast-related genes (*Trap, Ctsk, Nfatc1, Mmp9*) in BMM cells (n = 4). **f**) and **g**) Volcanic map and heat map showing the difference in protein expression between the Zn-0.8 Mg alloy group and control group. **h**) Parallel reaction monitoring quantitative assay verifying changes in the key proteins involved in osteoclast differentiation regulation by the Zn-0.8 Mg alloy evaluated by TMT proteomic analysis (n = 3).

After 14 days of induction, the Zn-0.8 Mg alloy extract group exhibited 1442 differentially expressed genes, with 779 upregulated and 663 downregulated (Fig. 2c). Osteogenesis-related genes (*Spp1, Ibsp, Dmp1, Omd, Bglap, Bglap2, Bglap3, Scube2, Phospho1,* and *Phex*) showed significant upregulation, as did *Mt1*, *Mt2* (Fig. 2e). Fig. 3d shows a significant increase in biomineral tissue development, biomineralization, bone mineralization, and other genes related to osteogenesis (indicated by the red frame), which explains the superior *in vitro* osteogenic characteristics of the Zn–0.8 Mg alloy (Fig. 2d). Additionally, KEGG analysis demonstrated significant enrichment in the Pi3k/Akt pathway, cell adhesion molecules, focal adhesion, and ECM-receptor interaction (Fig. 2f).

Notably, the Pi3k/Akt pathway is vital for facilitating osteoblast differentiation and represents a major mechanism promoting bone formation through various natural compounds, including microRNA, exosomes, and filipin/hydroxyapatite [[34], [35], [36]]. Similarly, we suggest that the Pi3k/Akt signaling pathway is a key contributor to osteogenic differentiation promoted by the Zn-0.8 Mg alloy extract following the longer period of induction (14 days). The degradation products of the Zn-0.8 Mg alloy activated the core genes of the Pi3k/Akt signaling pathway by upregulating signals such as the upstream genes *Spp1, Ibsp, Ifna13*, leading to elevated expression of *Runx-2, Omd, Bglap*, and other markers of osteogenic differentiation. Additionally, *Mt1/2* expression was notably elevated in the Zn-0.8 Mg group. As an intracellular metal-binding protein, the expression of *Mt1/2* was regulated by the intracellular Zn concentration, with elevated *Mt1/2* v expression in turn regulating the Zn concentration and promoting intracellular Zn homeostasis [37]. Elevated *Mt1/2* v expression can also increase Akt phosphorylation and promote the Pi3k/Akt pathway [38,39]. Therefore, *Mt1/2* may be an alternative mechanism for activating the Pi3k/Akt pathway. *In vitro* validation using qPCR technology confirmed the upregulation of key genes in the Pi3k/Akt pathway (*Pik3r1, Pik3ca, Akt*), osteogenesis-related genes (*Ibsp, Dmp1, Bglap, Bglap2, Bglap3, and Phex*), with over a three-fold increase, and *Mt1/2* (Fig. 2g).

Zinc, as a necessary element of bone, plays a positive part in promoting bone growth and maturation. Both binary and ternary Zn alloys comprising different alloying elements show stable osteogenic promotion effects [40,41]. The stimulation of bone formation by Zn–Mg alloys has been extensively studied; however, the mechanism of osteogenic differentiation induced by Zn–Mg alloys remains unclear [42,43]. Similar to Zn–Li and Zn–Sr alloys, we found that Zn-0.8 Mg alloys also promote osteogenesis by activating the Pi3k-Akt signaling pathway [16,44]. This suggests that Zn^2+^ probably has a leading impact on regulating the osteogenic differentiation of Zn alloy degradation products.

### 3.3 Influence and mechanism of Zn-0.8 Mg alloy on osteoclast differentiation and bone resorption *in vitro*

The mechanisms resulting in aberrant bone metabolism *in vivo* are complex and varied, with excessive bone resorption through osteoclast activation being key. Therefore, the ideal biological properties of implant materials used to treat fractures/bone defects in patients with aberrant bone metabolism should include an inhibitory effect on osteoclasts. Therefore, we further investigated the influence of Zn-0.8 Mg alloy on osteoclast differentiation. The number and area of osteoclasts were significantly decreased in the Zn-0.8 Mg alloy group in contrast to other two groups (Fig. 3a and b). The bone resorption of osteoclasts was further evaluated by culturing mononuclear macrophages on calf bone slices with osteoclast differentiation induction solution. SEM revealed that a plethora of bone resorption traps existed on the bone slices of the control group, whereas the area of pits was significantly decreased in the pure Zn group. However, almost no obvious bone resorption pits were observed after treatment with Zn-0.8 Mg extracts (Fig. 3c and d). qPCR assays were performed to analyze the expression of osteoclast-related genes to further clarify the effects of the Zn-0.8 Mg alloy on osteoclast differentiation. The results indicated that pure Zn markedly decreased the expression levels of *Trap, Ctsk, Nfatc1,* and *Mmp9*. Moreover, the above genes were further reduced following stimulation with Zn-0.8 Mg alloys (Fig. 3e). Furthermore, the Zn-0.8 Mg alloy demonstrated stronger inhibitory effect on osteoclasts than that of pure Zn. According to our previous study, the Zn^2+^ concentration in the extraction solution of the Zn-0.8 Mg alloy did not change significantly from that of pure Zn [24]. Thus, the synergistic impact of Zn^2+^ and Mg^2+^ was likely responsible for the superior osteoclast inhibition of the Zn-0.8 Mg alloy.

To elucidate this regulatory mechanism by which the Zn-0.8 Mg alloy inhibits osteoclast differentiation, we performed TMT quantitative proteomics to investigate differential protein expression compared to the blank control. Based on the criteria of the multiplicity of differences: fold change (FC) value > 1.4 or FC < 5/7 with P < 0.05, a total of 3683 plausible proteins were screened to obtain significant differential proteins. Proteomics revealed that 518 proteins were upregulated and 541 proteins were downregulated in the Zn-0.8 Mg alloy group (Fig. 3f and g). According to further GO and KEGG analyses and validation by PRM, the proteins related to osteoclast differentiation, GRB2, ERK, and *p*-ERK, were significantly reduced (Fig. 3h).

GRB2 is a key coupling junction protein. The SH2 structural domain of GRB2 binds to tyrosine phosphorylated by the growth factor receptor tyrosine kinase (RTK), whereas the SH3 structural domain exhibits an affinity for proline-rich sequences in the SOS protein [45]. By coupling RTK and SOS, Ras/Raf/MEK was activated sequentially. Finally, ERK was activated and phosphorylated. The phosphorylated ERK not only phosphorylated cytoplasmic proteins but also phosphorylated some intranuclear transcription factors, for example, c-fos and c-Jun, and thus participated in regulating osteoclast proliferation and differentiation. Previous studies have found that adjusting the elemental Zn content of rat diets can inhibit RANK expression by inhibiting ERK activation, thereby reducing osteoclast differentiation [46]. Moreover, high concentrations of Mg^2+^ can inhibit autophagy in the ATDC5 cell line by suppressing the phosphorylation of ERK [47]. Therefore, a synergistic effect may exist between the products of Zn-0.8 Mg alloy degradation, leading to inhibition of the GRB2/ERK signaling pathway, which ultimately results in a stronger suppressive effect on osteoclast differentiation.

The effect of zinc on osteoclast differentiation is influenced by dose [48,49]. Roy et al. found that Zn-doped tricalcium phosphate can significantly reduce the TRAP expression of osteoclasts *in vitro*, whereas doping both Zn and Sr had no significant difference, indicating that the osteoclast inhibition effect of zinc is affected by other metal elements [50]. Moreover, different Zn-based alloys exhibit different degradation rates. The composition and content of alloy elements are also diverse. This may explain the lack of reports regarding Zn-based alloys inhibiting osteoclast differentiation and proliferation. In previous research, we found that the Zn–Ag alloy effectively suppresses osteoclast differentiation both *in vitro* and *in vivo*; however, no further studies have elucidated the mechanism [17]. In this study, the key signaling pathway by which the Zn–Mg alloy inhibits osteoclast differentiation was explored by proteomics technology, which clarified the synergistic regulation of osteoclasts by Zn^2+^ and Mg^2+^, highlighting the substantial potential of this alloy for bone repair.

### 3.4 Zn-0.8 Mg alloy inhibits wear particle-induced calvarial osteolysis

**Fig. 4:**
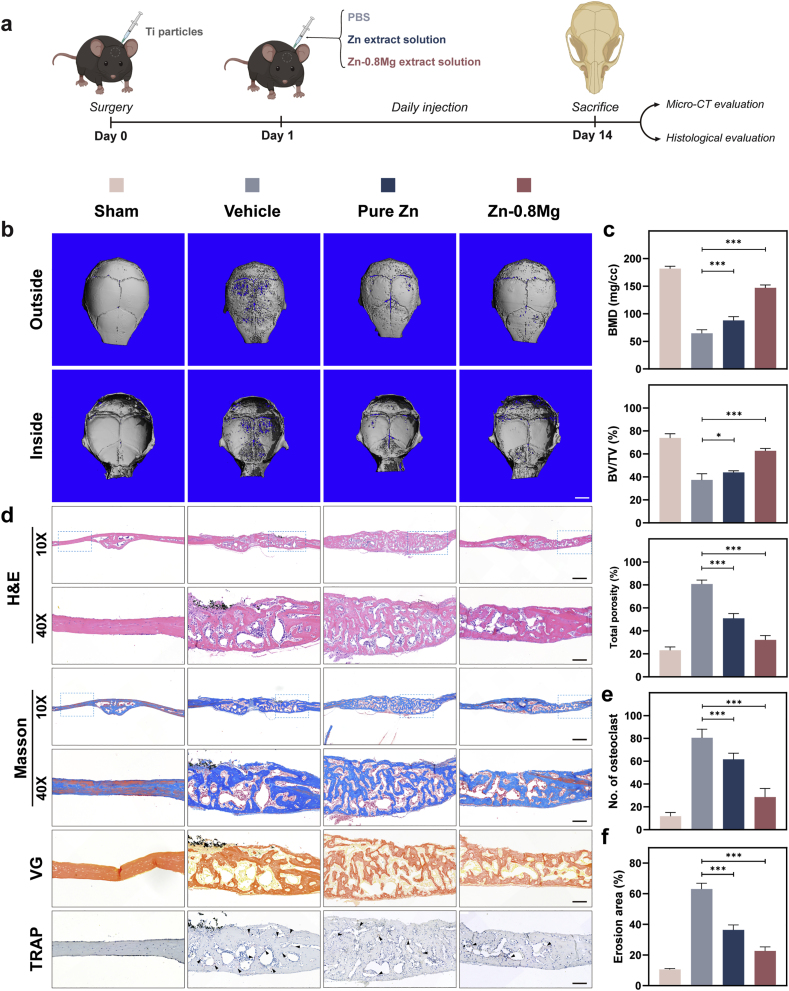
Zn-0.8 Mg alloy inhibited osteoclast activation in a mouse calvarial osteolysis model. **a**) Experimental outline of mouse cranial osteolysis model construction. **b**) and **c**) Micro-CT images of the skull at 14 days (**b**) and quantification of bone mineral density (BMD), bone volume per total volume (BV/TV), and total porosity (**c**) in the mouse cranial bone (n = 5). Scale bar: 2 mm. **d**) and **e**) Representative H&E, Masson, Van Gieson (VG), and TRAP staining images. TRAP-positive osteoclasts are indicated by black arrows. **e**) and **f**) Quantitative analysis of osteoclast numbers and bone resorption area from TRAP immunohistochemistry staining (n = 5). Scale bar: 500 μm (10×), 200 μm (40×).

*In vivo*, osteoclast differentiation is an intricate regulatory process influenced by multiple factors such as growth factors and cytokines, including tumor necrosis factor (TNF) secreted by immune cells, OPG secreted by osteoblasts, and M-CSF [[51], [52], [53]]. Therefore, we constructed a Ti particle-mediated mouse cranial osteolysis model to simulate the over differentiation and activation of osteoclasts stimulated by aseptic inflammation, as well as the complex effects of Zn-0.8 Mg extraction solution on osteoclasts in an elaborate environment *in vivo* (Fig. 4a). Micro-CT results indicated that the cranial surfaces of the sham operation group were smooth and flat with no obvious bone resorption (Fig. 4b). In contrast, following the addition of Ti particles to the vehicle group, we observed a wide range of bone resorption holes and fractures on the cranial surfaces and sutures, respectively. After local treatment with pure Zn extract, osteolysis was suppressed, and the extent of bone resorption on the skull surface was significantly decreased. In contrast, local application of Zn-0.8 Mg extract led to stronger suppression of osteolysis. Except for a further reduction in the extent of bone resorption, no obvious fractures were observed at the cranial suture. The quantitative results of bone mineral density (BMD), bone volume per total volume (BV/TV), and porosity agreed with trends observed in the micro-CT images (Fig. 4c). The Zn-0.8 Mg extract exhibited greater inhibition of osteolysis than other treatments.

The obtained cranial samples were subjected to histomorphometry analysis, including H&E staining, TRAP, Masson, and Van Gieson (VG) staining, to further assess the effect on osteolysis (Fig. 4d). Ti particles observed by H&E staining led to significant bone destruction and a large infiltration of inflammatory cells and fibrous tissue in the dissolved bone. Notably, the region of inflammatory soft tissue infiltration was reduced in the Zn-0.8 Mg alloy group compared to the other two groups. The morphology and number of osteoclasts were further observed by TRAP staining; many TRAP-positive osteoclasts were present in the Ti particle group, whereas osteoclasts were significantly decreased in the Zn-0.8 Mg group (Fig. 4e). Additionally, abnormal osteoclast activation sites were often accompanied by active bone metabolism, causing a rise in the quantity of immature bone, with both Masson and VG staining suggesting that the sham group had mature bone, whereas the Ti particle group had predominantly immature bone, despite being thicker; bone maturity of Zn-0.8 Mg alloy group was intermediate between these two groups. These results indicate that Zn–Mg alloy degradation products can effectively suppress osteoclast activation *in vivo*.

### 3.5 Zn-0.8 Mg alloy promotes the bone healing of femoral condyle fractures in rabbits

**Fig. 5:**
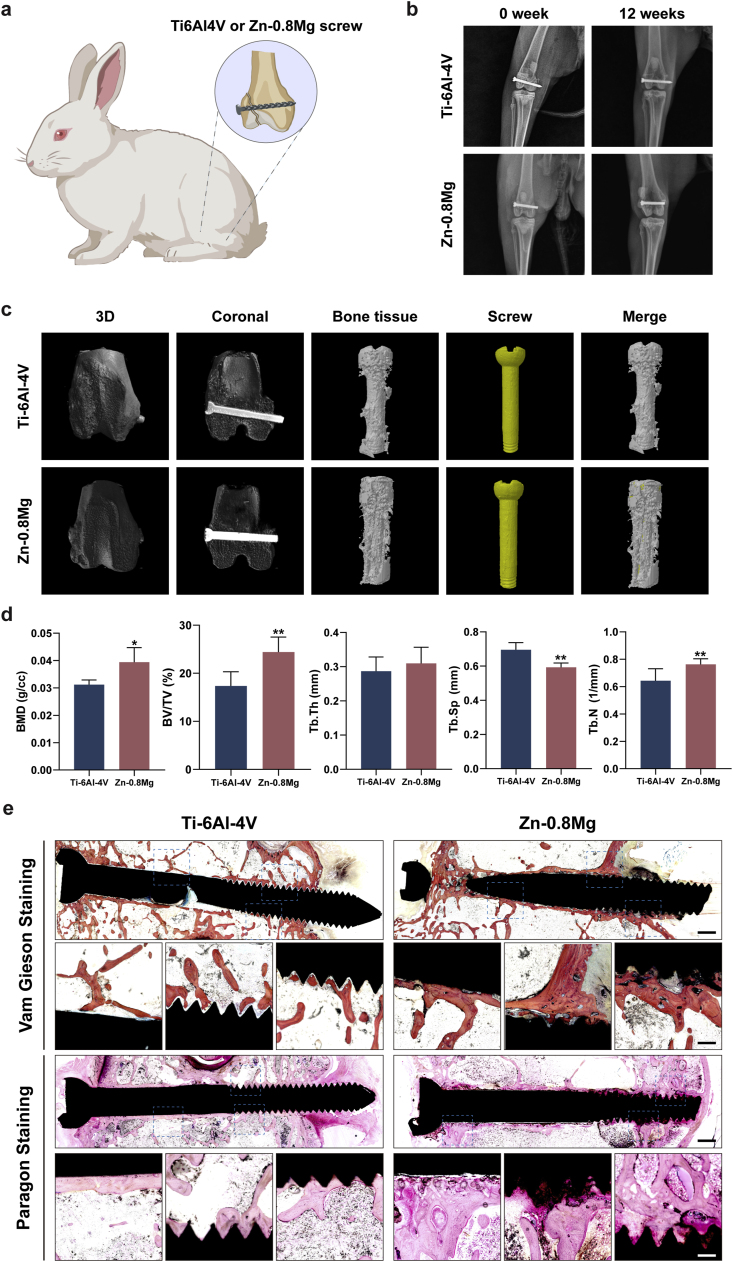
Zn-0.8 Mg alloy demonstrated superior internal fixation and enhanced fracture healing in a rabbit femoral condyle fracture model. **a**) Experimental illustration of internal fixation in the rabbit femoral condyle fracture model. **b**) Representative radiographic results showing femurs post-operation and three months post-operation. **c**) Micro-CT images of femurs three months post-operation, and **d)** quantification of BMD, BV/TV, trabecular thickness (Tb·Th), trabecular separation (Tb.Sp), and trabecular number (Tb·N) in rabbit femurs (n = 5). **e**) Representative Van Gieson and Paragon staining of rabbit femoral tissue sections. Scale bar: 1 mm, 200 μm (enlargement).

It is worth noting that osteoclasts and osteoblasts can interact with each other through direct contact, secretion of paracrine factors, and interaction between cells and the bone matrix to regulate their differentiation and biofunction [54]. Therefore, based on the synergistic effect of the Zn-0.8 Mg *in vitro* extract in regulating bone metabolism from both directions, we designed a femoral condyle fracture rabbit model to assess the overall efficacy of the Zn-0.8 Mg alloy as a common orthopedic implant (internal fixation screw) to promote bone healing *in vivo* (Fig. 5a). Radiography performed immediately post-surgery showed that the femoral condyles of both groups had a distinct fracture line, and the screws effectively fixed the fracture ends (Fig. 5b). Three months after surgery, radiographic results demonstrated that the femoral condyles of both groups exhibited a good position and the fracture lines had disappeared, indicating good fracture healing (Fig. 5b). Micro-CT images taken three months post-surgery also showed that the fractures in both groups had healed without obvious fracture lines (Fig. 5c). Moreover, we observed an obvious improvement in new bone tissue around the Zn-0.8 Mg screws, unlike in the Ti alloy group. Surgical areas and surrounding bone were subjected to quantitative analysis. The results showed that, BMD, BV, BV/TV, and trabecular number (Tb·N) were significantly higher in the Zn-0.8 Mg group than in the Ti–6Al–4V group (Fig. 5d). This indicated a larger bone volume, a higher bone density, and an elevated count of trabeculae. Conversely, trabecular separation (Tb.Sp) was significantly decreased in the Zn-0.8 Mg group, underscoring a higher bone density in the former. In addition, trabecular thickness (Tb·Th) showed no statistically significant difference between the two groups.

**Fig. 6:**
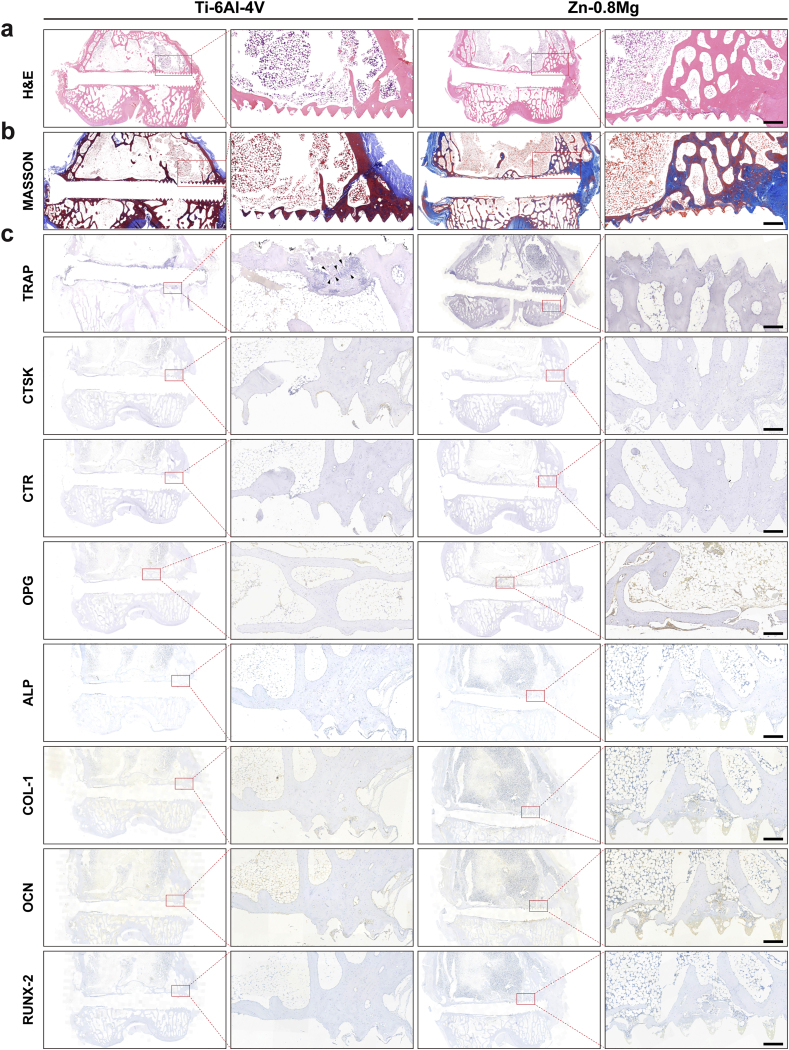
Representative staining images of femoral tissue sections three months post-operation. H&E staining (**a**) and Masson staining of rabbit femoral tissue sections (**b**). **c**) Immunohistochemical staining of osteoclast-related (TRAP, CTSK, CTR) and osteogenic (OPG, ALP, COL-1, OCN, RUNX-2) genes. Red boxes represent local zoom areas. Scale bar: 1 mm, 200 μm.

Femoral condyle hard tissue sections of both groups three months post-surgery were obtained and subjected to VG and Paragon staining (Fig. 5e). Compared to the Ti–6Al–4V group, VG-stained sections showed multiple degradation products and newly regenerated bone surrounding the Zn-0.8 Mg screws, as well as greater osseointegration on the surface of the screw in the Zn-0.8 Mg group. The bone highlighted in pink by Paragon staining was also significantly increased around the Zn-0.8 Mg screw and tightly integrated with the surface of the screw. Further tissue sections were perfected using H&E and Masson staining, which revealed that both groups were effective in repairing rabbit femoral condyle fractures (Fig. 6a and b).

Osteoblast and osteoclast activities are key factors affecting bone healing in patients with fractures/bone defects combined with aberrant bone metabolism. Thus, immunohistochemical staining was conducted to investigate the mechanism of osteointegration in the Zn-0.8 Mg screw group (Fig. 6c). Osteogenesis-specific matrix proteins include ALP, OPN, and COL I, with RUNX-2 being the most widely recognized osteoblast nuclear transcription protein [55]. These four osteogenesis-related marker proteins showed significant positive staining in tissues surrounding the Zn-0.8 Mg alloy screws, unlike in the Ti–6Al–4V alloy group. For osteoclasts, osteoclast-specific marker enzyme staining (TRAP, CTSK, CTR) was clearly observed around the Ti–6Al–4V screws, whereas fewer positive staining areas were observed surrounding the Zn-0.8 Mg screws. OPG can efficiently bind to RANKL or TRAIL, blocking the association between RANKL or TRAIL and its corresponding transmembrane receptors, and regulates the RANKL/RANK pathway to influence osteoclast differentiation [56]. High expression of OPG was detected around the Zn-0.8 Mg screws, unlike in the Ti–6Al–4V screw group. These observations are compatible with the significant osteoclast inhibitory influence of the Zn-0.8 Mg extraction solution detected in the mouse calvarial osteolysis model, suggesting that both the *in vitro* extract and *in vivo* environment of the degradation products are capable of exerting an inhibitory effect on osteoclasts. Overall, the above results further validated the pro-integration mechanism of Zn-0.8 Mg screws in inducing osteogenic differentiation and inhibiting osteoclast activity.

The importance of the interactions between the immune and skeletal systems has received widespread attention and gradually evolved into osteoimmunology [57]. Disorders of the immune system are also important causes of bone metabolism disorders. Therefore, the mutual reaction between the bone immune microenvironment and the degradation products of the implant is a key factor that should be considered when evaluating implants. In contrast to the Ti–6Al–4V screw group, no significant positive staining was observed for proinflammatory indicators (IL-1β, TNF-α, IL-6) in the Zn-0.8 Mg screw group, whereas a significant positive area was observed for the anti-inflammatory cytokine (IL-10), which suggests that Zn-0.8 Mg alloys have good biocompatibility *in vivo* (Fig. S4). As the dominant immune cells in the circulatory system, neutrophils are believed to serve as killers of foreign matter in the innate immune system. After the alloy is implanted as a foreign body, neutrophils first reach the site of inflammation and activate and recruit macrophages. Previous studies have mentioned the direct activation of neutrophils by biomaterials, resulting in the release of reactive oxygen species and neutrophil extracellular traps (NETs) [58]. Therefore, neutrophils may be a key factor affecting Zn-0.8 Mg alloy performance in our study. LPS and phorbol-12-myristate-13-acetate all both broad-spectrum cellular simulators, which can activate the formation of NETs [59,60]. However, as the most common model of inflammation *in vitro*, LPS stimulation can activate immune cells to release inflammatory cytokines and chemokines and more closely resemble the immune response state, whereas phorbol-12-myristate-13-acetate does not have this capability [61,62]. LPS was consequently used to simulate the inflammatory environment *in vitro*; the expression of proinflammatory cytokines (*Il-1β, Tnf-α, Il-6*) and NET-related genes (*Pad 4, Ne*) was significantly downregulated after treatment with Zn-0.8 Mg extract in contrast to the control group (Fig. S6). Conversely, the expression of calprotectin (*S100a8, S100a9*) and chemokines (*Cxcl10*) was significantly upregulated. Therefore, we suggest that the effect of Zn-0.8 Mg on neutrophils may be important for reducing inflammation during implantation.

Additionally, we noted not only an absence of osteolysis or dislocation of the implant but also no obvious inflammatory reaction around the implant. In the fracture model, three months after Zn-0.8 Mg screw implantation, no obvious pathological changes were detected in H&E-stained sections of rabbit distant organs (heart, liver, spleen, lung, and kidney), suggesting that the degradation products have no obvious toxicity to normal local and systemic tissues, which emphasizes the good biocompatibility of Zn-0.8 Mg screws (Fig. S7).

In conclusion, the Zn-0.8 Mg alloy demonstrates significant superiority over the Ti alloy in promoting bone healing and regeneration in rabbit femoral condylar fracture models. Although no significant difference was observed in the fracture healing images of the two alloys, the biodegradability and bidirectional regulatory ability of bone metabolism are the key advantages of the Zn-0.8 Mg alloy compared to the inert metal materials commonly used in clinics. Moreover, the biodegradable nature of it eliminates the need for a second removal operation. Hard tissue sections indicated degradation of the Zn-0.8 Mg alloy, but minimal change in its overall shape three months post-implantation, suggesting that it maintained mechanical integrity as an internal fixation screw during the critical period of fracture healing (3–6 months). Furthermore, immunohistochemical results demonstrated bidirectional regulation of bone resorption and formation, highlighting its potential for healing fractures and bone defects in patients with abnormal bone metabolism.

### 3.6 3D-printed Zn-0.8 Mg alloy scaffold accelerates bone defect repair in aged postmenopausal rats

**Fig. 7:**
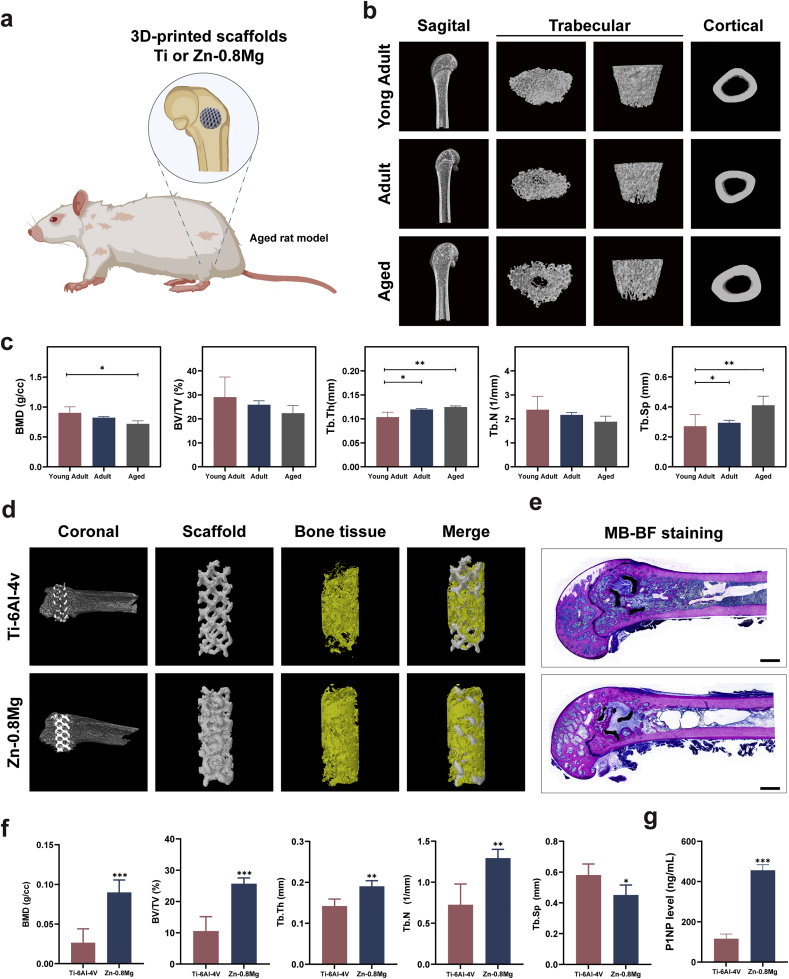
3D-printed Zn-0.8 Mg alloy scaffold promoted the femoral condyle defect repair in aged postmenopausal rats. **a**) Experimental illustration of the rat femoral condyle defect model. **b**) and **c**) Representative micro-CT images of rat femurs across various age groups (**b**) and quantification of BMD, BV/TV, Tb·Th, Tb. Sp, and Tb·N (**c**) (n = 5). **d**) Micro-CT images of rat femurs three months post-surgery. **e**) Methylene blue-basic fuchsin staining highlighting new bone (red areas) and fibrous tissue (blue areas). **f**) Quantification of BMD, BV/TV, Tb·Th, Tb. Sp, and Tb. In aged rat femurs (n = 5). **g**) Quantitative assessment of type I collagen amino-terminal prolongation peptide in serum (n = 5). Scale bar: 1 mm.

The Zn–Mg alloy demonstrated excellent bidirectional bone metabolism-modulating effects as well as improved osteointegration *in vivo*, thereby meeting the requirements of an ideal implant material for healing fracture/bone defects in patients with bone metabolism abnormalities. Therefore, we constructed a model of femoral condyle defects in aged postmenopausal rats to further emphasize the potential of Zn-0.8 Mg for specific applications in clinically refractory diseases (Fig. 7a). Although the rat osteoporosis model constructed by ovariectomy is the most common animal model for studying abnormal bone metabolism as it effectively simulates the characteristics of postmenopausal bone metabolism, it may not be completely consistent with clinical practice [63]. Patients with postmenopausal osteoporosis exhibit low ovarian function, but ovarian stromal cells still show certain endocrine function after menopause [64]. Furthermore, the bones of rats continue to grow throughout their lives. Therefore, when young rats are selected for castration models, the bones are not mature and bone remodeling is lacking [65]. In addition, patients with postmenopausal osteoporosis not only face increased bone resorption caused by decreased estrogen secretion, but also decreased metabolism and activity levels of various cells. The status of estrogen in the pathogenesis of postmenopausal osteoporosis has been challenged by aging and reactive oxygen species [66]. However, an aged female rat model can fully simulate the effects of aging and estrogen. Therefore, we selected 24-month-old rats as experimental animals to challenge the bone metabolic regulation of Zn-0.8 Mg. The 24-month-old rats, equivalent to humans at approximately 60 years of age, are already in the reproductive senescence stage [[67], [68], [69]]. Aged females are the most susceptible to osteoporosis, as ovarian function gradually declines and a decrease in estrogen levels leads to an increase in RANKL levels, which induces osteoclast differentiation and accelerates bone decomposition [70]. Simultaneously, the function of osteoblasts and bone formation decreases in the aging state, triggering an imbalance in the coupling of bone formation and resorption. Aged postmenopausal osteoporosis is characterized by decreased bone density, increased porosity, and destruction of the trabecular structure of cancellous bone [71]. At its core, bone loss is caused by an imbalance in the bone-remodeling cycle; both the bone-remodeling cycle and turnover are lower for cancellous bone than for cortical bone [72]. Therefore, fractures/bone defects are more likely to occur in the trabecula-rich metaphyseal region than in the diaphysis of the long bone [73,74]. This is consistent with our results on bone density and other related indices of rat femurs across various age groups measured by micro-CT (Fig. 7b). The results demonstrated a significant increase in the BMD of cancellous bone in aged rats, but a significant decrease in the Tb·Th and Tb. Sp in contrast to those of young and adult rats (Fig. 7c). This indicates reduced bone volume and bone trabecula density. In addition, analysis of the cortical bone region indicated no significant change in the cortical bone volume or porosity in aged rats (Fig. S8).

In addition, 3D printing technology has a clear advantage in reconstructing bone defects as it can adapt the shape and dimensions of a specific bone defect in an efficient and stable manner. Furthermore, the printed porous scaffold structure facilitates inward angiogenesis and bone growth. The mechanical characteristics and degradation rate of the implant can be controlled by regulating porosity [75]. Previously, we reported the excellent effect of pure Zn and Zn–1Mg porous scaffolds constructed by additive manufacturing to repair common rabbit femoral bone defects [76,77]. However, this is the first attempt to use an additive zinc alloy scaffold to treat an animal model of bone defects with abnormal bone metabolism. In the process of bone defect repair, the pore structure of the scaffold provides space for the growth of osteoblasts and interaction between cells [78]. Scaffolds with high porosity possess a larger surface area, facilitating enhanced interaction with the ECM and supporting the internal growth of new bone and blood vessels [79]. In addition, prolonged interaction between the scaffold and the ECM will contribute to pore occlusion. Thus, it is important to ensure the effective penetration of nutrients and biomolecules within the scaffold by maintaining a high porosity. The porosity of human cancellous bone is 50–90 %. Considering the bionic bone structure, this represents the ideal parameter range for bone implantation scaffolds [80,81]. Although higher porosity can promote higher bone inward growth, this comes at the expense of the mechanical energy of the scaffold [82]. Here, the compressive strength of the Zn-0.8 Mg alloy scaffold with 80 % porosity was 7.43 ± 0.62 MPa, which was within the compressive strength range of cancellous bone (2–12 MPa), meaning that the proposed alloy can provide adequate mechanical support for bone defects of the femoral condyle. In our previous study, 80%-porosity Zn–2Mg scaffold exhibited better biocompatibility and osteogenesis promotion [43]. Therefore, to evaluate the therapeutic effect of Zn-0.8 Mg scaffolds on bone defects with low bone metabolism status, 80%-porosity of scaffolds were selected.

Three months after implantation, rat femurs were acquired for micro-CT analysis. The Zn-0.8 Mg scaffold group exhibited a higher rate of inward bone growth compared with Ti–6Al–4V scaffold group, revealing the better bone regeneration-inducing activity of Zn-0.8 Mg scaffolds (Fig. 7d). In the case of fractures and bone defects, the implants were preserved for at least 3–6 months for mechanical support [83]. In the three-month *in vivo* experiments, the stents in the Ti–6Al–4V group showed no visible volume or shape changes. Limited biodegradation products were detected surrounding the Zn-0.8 Mg scaffold group. However, the porous scaffold structure remained intact (Fig. 7d), suggesting that the Zn-0.8 Mg alloy provides consistent support to the bone. However, the Zn-0.8 Mg alloy must be tested *in vivo* over a longer term to observe intact degradation behavior.

Quantitative assessment of the newly regenerated bone quality at the defect site indicated that the BMD, BV, BV/TV, and Tb·N were markedly increased in the Zn-0.8 Mg scaffold group compared with the Ti–6Al–4V scaffold group. A significant decrease of Tb·Th and Tb. Sp were observed in the Zn-0.8 Mg scaffold group (Fig. 7f). Tissues were obtained and stained with hard tissue methylene blue-basic fuchsin staining; new bone tissue was red, whereas fibrous tissue was blue (Fig. 7e). The results revealed that more new bone grew in the internal pores of the 3D-printed Zn-0.8 Mg alloys, and the quantity of bone around the material was also notably higher. Thus, compared with Ti–6Al–4V, Zn-0.8 Mg displayed remarkably osseointegration in the bone defects of an aged postmenopausal animal model. Additionally, the total type I collagen amino-terminal prolongation peptide was notably elevated in the Zn-0.8 Mg scaffold group (Fig. 7g). This peptide is a specific marker of type I collagen deposition and serves as a bone formation marker to detect osteoblast viability and bone formation [84]. As described above, the results indicated that the Zn-0.8 Mg alloy is able to improve bone defect repair in the aberrant bone metabolism environment of senescent cell hypofunction and estrogen-stimulated osteoclast activation, suggesting its potential as a new strategy for treating fractures/bone defects in patients with aberrant bone metabolism.

**Fig. 8:**
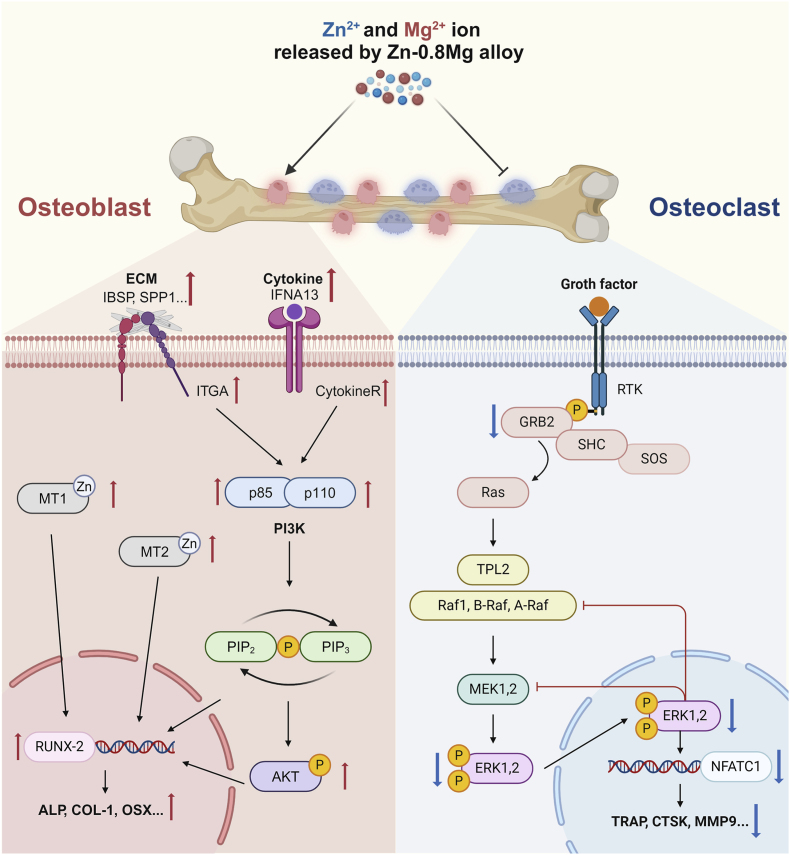
Zn-0.8 Mg alloy may exert bidirectional regulation on osteoblasts and osteoclasts through the Pi3k/Akt and GRB2/ERK signaling pathways, respectively. Schematic created with BioRender.com (Agreement number: ML26FL6QFY).

Current treatment strategies for fractures/bone defects in patients with aberrant bone metabolism prefer biomaterials loaded with osteoporosis therapeutic agents to modulate bone metabolism. For example, Che et al. utilized a bionic scaffold that promoted bone formation by delivering teriparatide [85]. However, the ability to maintain long-term effective and safe dose release and the complexity of the material preparation process limit the clinical application of drug-loaded biomaterials. In this study, starting from the basic materials of implants, we developed a Zn–Mg alloy material that can release Zn^2+^ and Mg^2+^, two essential metal ions, through good degradation behavior. The Zn-0.8 Mg alloy plays a bidirectional regulatory role in promoting osteogenesis and inhibiting osteoclast differentiation through the Pi3k/Akt and GRB2/ERK pathways, respectively (Fig. 8). Moreover, Zn–Mg alloys can be used to prepare implants with various morphologies for personalized customization using 3D printing technology, which has the benefits of low cost, high efficiency, and structurally specific adaptation. In summary, the Zn-0.8 Mg alloy shows excellent potential for clinical applications, especially for promoting bone healing in patients with fractures and defects combined with aberrant bone metabolism.

## 4 Conclusion

This study revealed that biodegradable Zn-0.8 Mg alloys have bidirectional functions in regulating bone metabolism. The Zn-0.8 Mg alloy not only promotes the proliferation and osteogenic differentiation of MC3T3-E1 cells by activating the Pi3k/Akt pathway, it also inhibits the GRB2/ERK pathway, which significantly affects osteoblastic differentiation and bone resorption in BMMs cells. We demonstrated that the Zn-0.8 Mg alloy inhibits the abnormal activation of osteoclasts induced by Ti particles in a mouse cranial osteolysis model. We also confirm its potential as an implant for treating conventional orthopedic diseases by verifying its ability to internally fix fractures and promote bone repair using Zn-0.8 Mg alloy screws in a rabbit femoral condyle fracture model. Based on these results, we prepared porous Zn-0.8 Mg alloy scaffolds using 3D printing technology to effectively treat femoral condyle defects in senescent postmenopausal rats through the bidirectional regulation of bone metabolism. In conclusion, because of its outstanding bidirectional regulation of bone metabolism, the Zn-0.8 Mg alloy demonstates clinical application potential for the specific treatment of fractures/defects in patients with abnormal bone metabolism.

## Ethics approval and consent to participate

All animal experiments adhered to the animal life protection standards and procedures sanctioned by Shanghai Rat & Mouse Biotech Co., Ltd (Issue No. 20230213 (14)).

## Data availability statement

The data that support the findings of this study are available from the corresponding author upon reasonable request.

## CRediT authorship contribution statement

**Jialian Xu:** Writing – original draft, Methodology, Investigation, Data curation, Conceptualization. **Guo Bao:** Writing – original draft, Methodology, Investigation, Funding acquisition, Conceptualization. **Bo Jia:** Writing – original draft, Methodology, Investigation, Data curation. **Minqi Wang:** Software, Methodology, Investigation, Data curation. **Peng Wen:** Validation, Methodology. **Tianyou Kan:** Methodology, Investigation. **Shutao Zhang:** Formal analysis, Data curation. **Aobo Liu:** Software, Formal analysis. **Haozheng Tang:** Methodology, Investigation, Formal analysis. **Hongtao Yang:** Writing – review & editing, Writing – original draft, Methodology, Investigation. **Bing Yue:** Writing – review & editing, Supervision, Funding acquisition. **Kerong Dai:** Supervision, Resources, Project administration, Methodology, Funding acquisition. **Yufeng Zheng:** Writing – review & editing, Supervision, Resources, Project administration, Methodology, Funding acquisition. **Xinhua Qu:** Writing – review & editing, Writing – original draft, Validation, Supervision, Funding acquisition, Conceptualization.

## Declaration of competing interest

Yufeng Zheng is an Editor in Chief for Bioactive Materials and was not involved in the editorial review or the decision to publish this article. Peng Wen and Xinhua Qu are editorial board members for Bioactive Materials and ware not involved in the editorial review or the decision to publish this article. All authors declare that there are no competing interests.
