# Supplementary material for: An adaptive biodegradable zinc alloy with bidirectional regulation of bone homeostasis for treating fractures and aged bone defects
Source: Bioact Mater. 2024 May 6;38:207–24. doi: 10.1016/j.bioactmat.2024.04.027 (PMC11096722; doi:10.1016/j.bioactmat.2024.04.027)
Supplement: Multimedia component 1 [file mmc1.pdf]

Supplementary Information

An Adaptive Biodegradable Zinc Alloy with Bidirectional Regulation of Bone Homeostasis for Treating Fractures and Aged Bone Defects

Xu et al.

Supplementary Figure

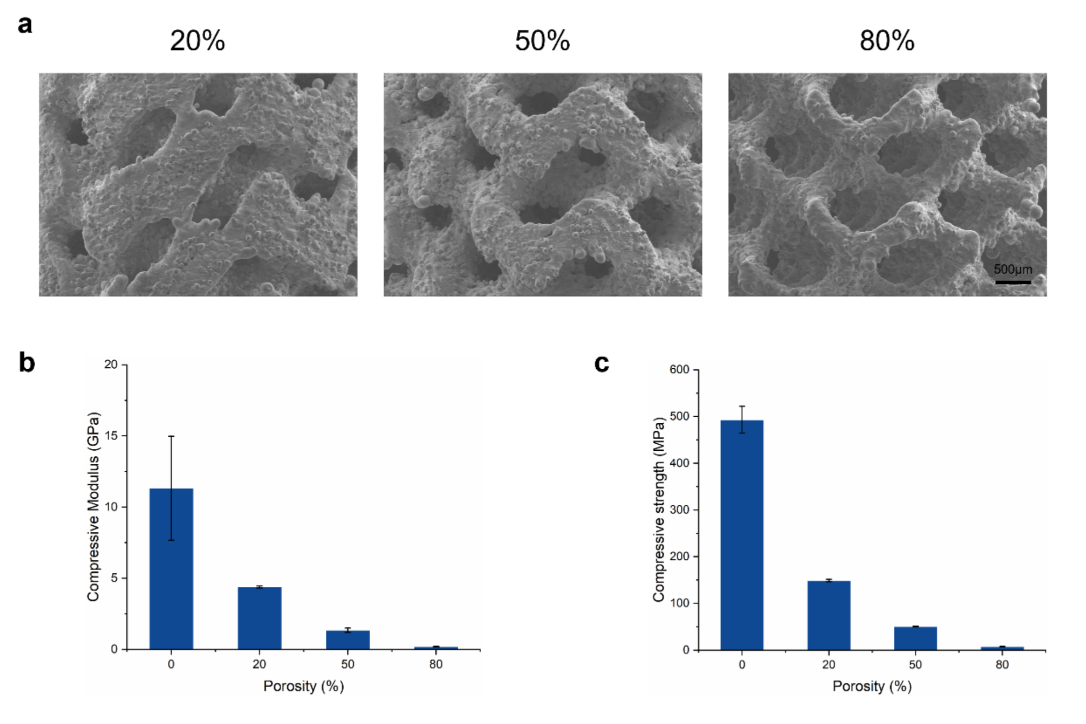

Figure S1. **a** SEM images of Zn-0.8Mg alloy scaffolds with different porosity (20%, 50%, 80%). Scale bar, 500μm. **b, c** Compressive test.

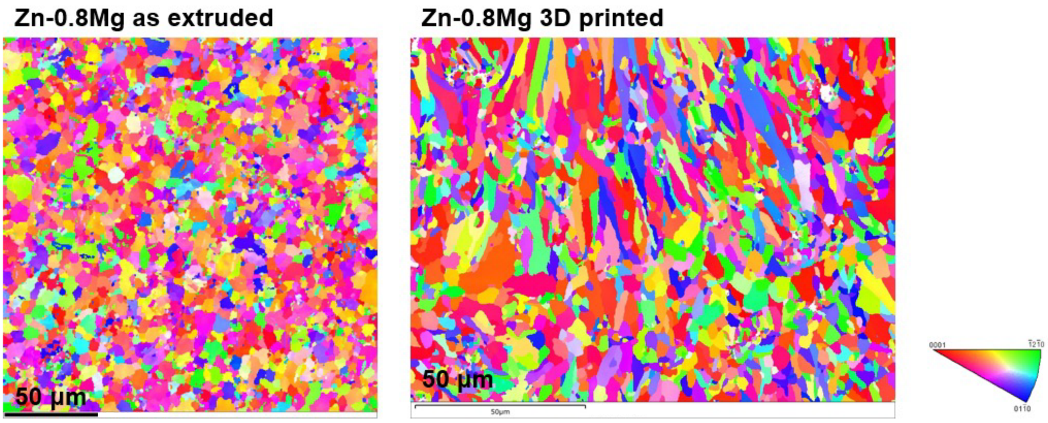

Figure S2. The microstructure of extruded and printed Zn-0.8Mg alloy

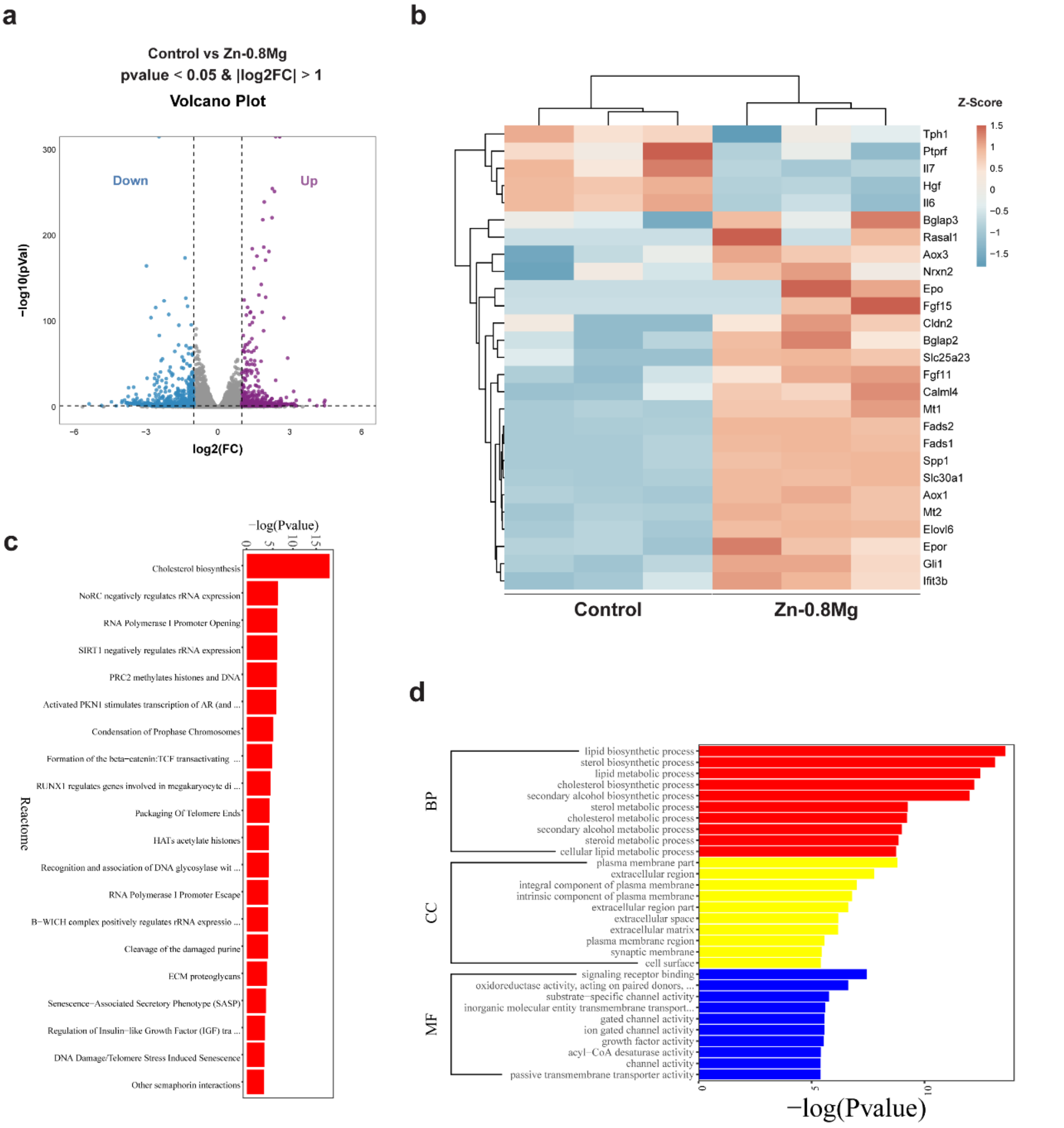

Figure S3. a) Volcano map and heat map showed the differential mRNA expression of MC3T3-E1 cells after 7 days culture. Purple and blue dots respectively represented up-regulated and down-regulated genes. **d**) and **f**) Enrichment analysis results of the GO and KEGG pathways. Red box highlights the important pathways that may be involved in the regulation of osteogenic differentiation of Zn-0.8Mg alloy. **g**) qPCR to verify the changes of key genes in regulating osteogenic differentiation of Zn-0.8Mg alloy obtained by RNA-seq analysis.

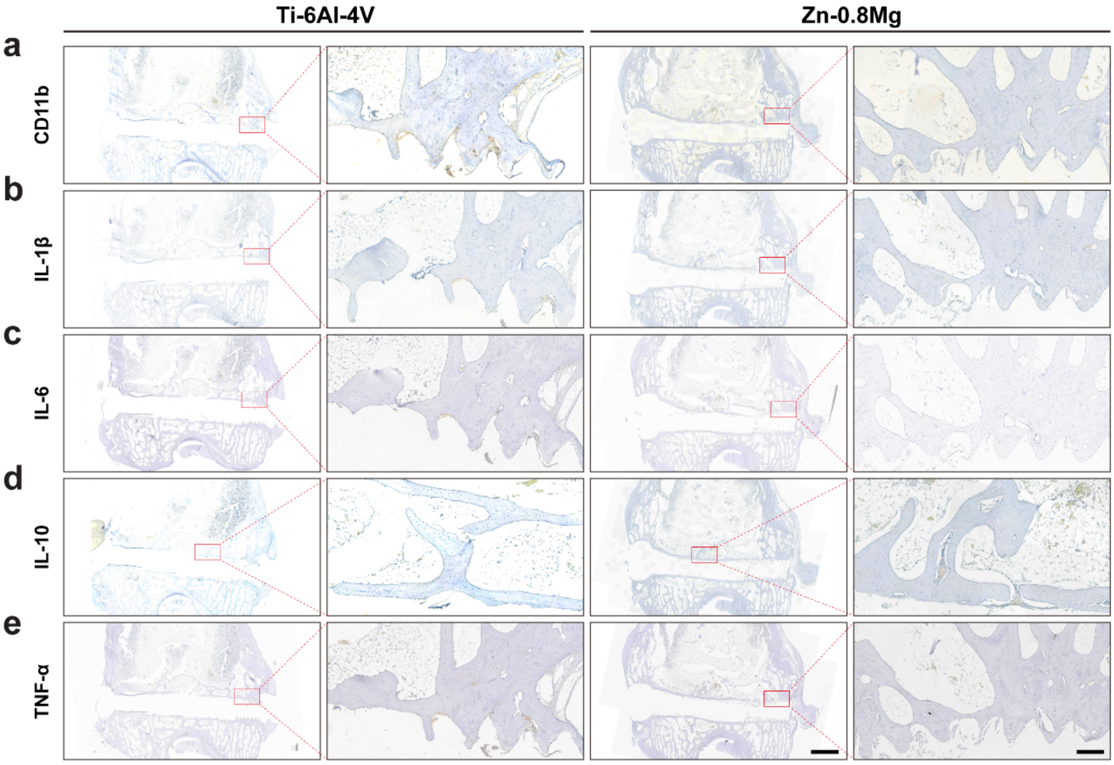

Figure S4. Representative immunohistochemistry staining of *CD11b, IL-1β*，*IL-6, IL-10, TNF-α* in rabbit femoral tissue sections at 3 months post-operation. Scale bar: 1mm, 200μm.

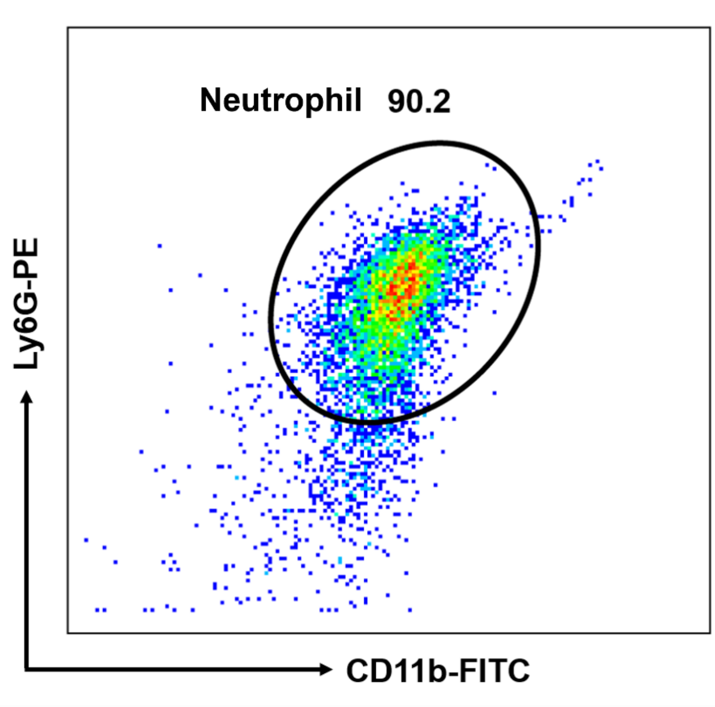

Figure S5. Purities of neutrophil isolated from bone marrow of C57BL/6 mice.

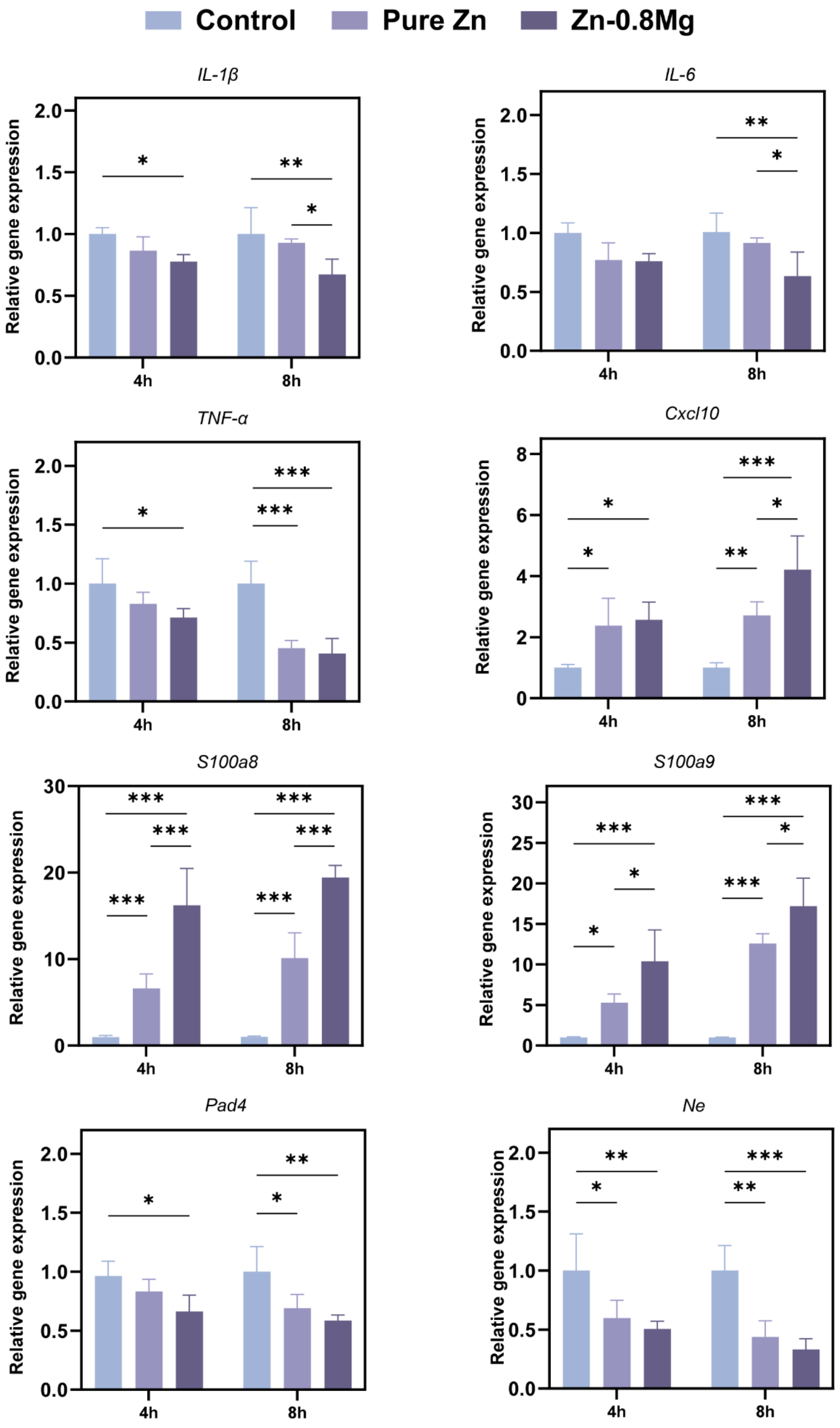

Figure S6. mRNA expression of *IL-1β*，*IL-6, TNF-α, Cxcl10, S100a9, S100a8, Pad4 and NE* in neutrophil treated with 100 ng/mL LPS for 4 hours and 8 hours. (*: $p<0.05$; **: $p<0.01$; ***: $p<0.001$)

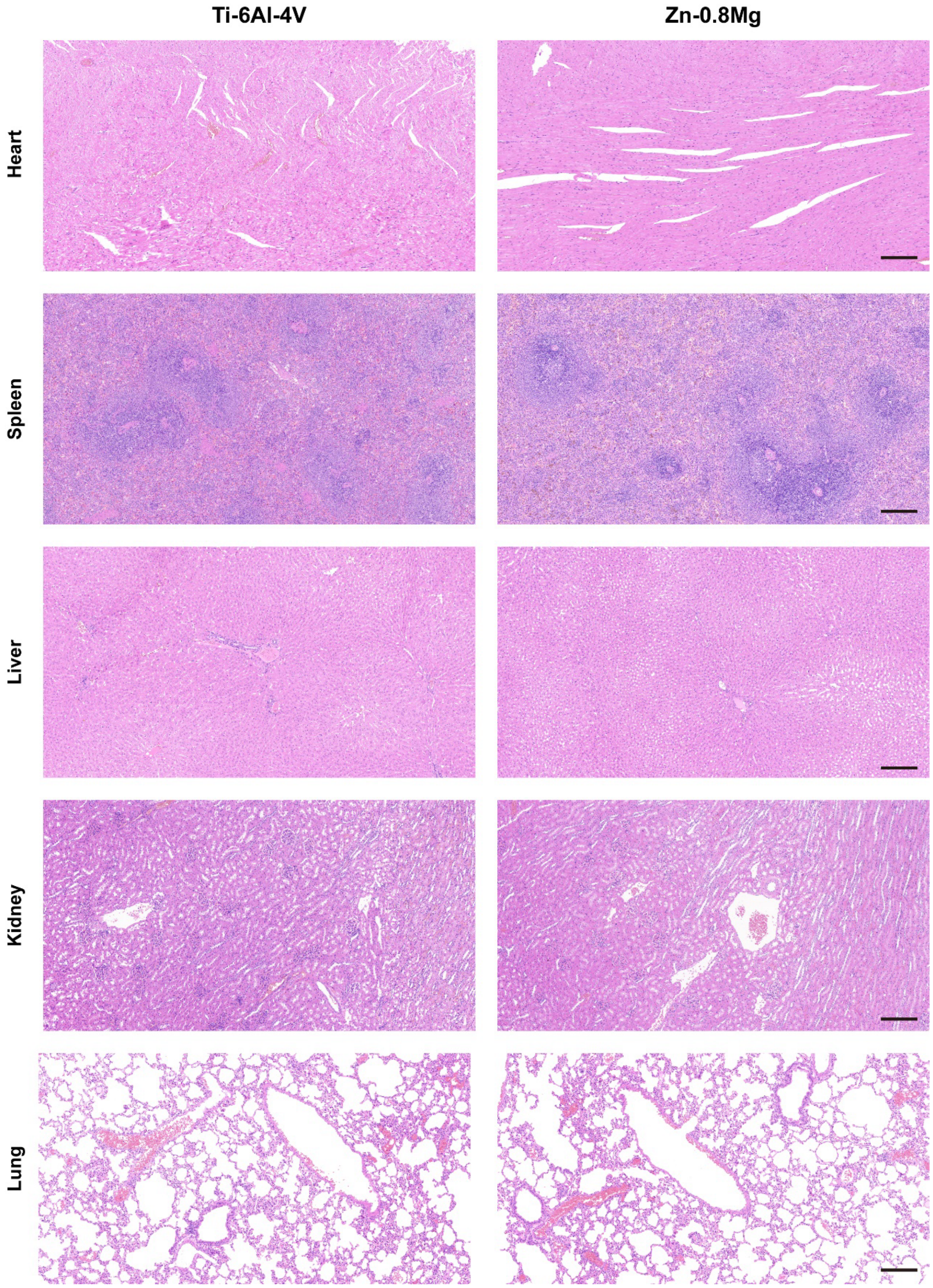

Figure S7. Representative H&E staining of rabbit organ sections at 3 months post-operation.

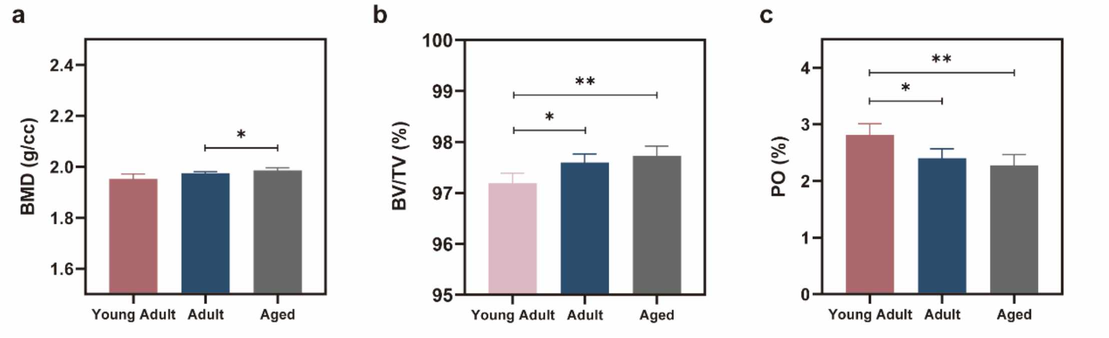

Figure S8. Micro-CT quantification of bone mineral density (BMD), bone volume per total volume (BV/TV) and total porosity (PO) in the rat femurs across various age groups.

Supplementary Table

Table S1. Primer sequence of MC3T3-E1 cells.

Genes		Primers（5′-3′）
β-actin	F	AGGTCCTCAGCTACAAGGAAG
	R	TCTTGAAGTCACAATCCTCTGGT
Alp	F	GGAGATGGTATGGGCGTCTC
	R	GGACCTGAGCGTTGGTGTTA
Col-1	F	TTCTCCTGGCAAAGACGGAC
	R	CTCAAGGTCACGGTCACGAA
Runx-2	F	TCGGAGAGGTACCAGATGGG
	R	AGGTGAAACTCTTGCCTCGT
Osx	F	ATGGCGTCCTCTCTGCTTG
	R	TGAAAGGTCAGCGTATGGCTT
Pik3r1	F	ACACCACGGTTTGGACTATGG
	R	GGCTACAGTAGTGGGCTTGG
Pik3ca	F	CCACGACCATCTTCGGGTG
	R	ACGGAGGCATTCTAAAGTCACTA
Akt	F	GTGTGGCAGGATGTGTATGAGAAG
	R	AGGCGGCGTGATGGTGATC
Ibsp	F	GCGACGAGGAAGAAGAAGAAGAAG
	R	TGGTGCTGGTGCCGTTGAC
Dmp1	F	AAGACTGTCATTCTCCTTGTGTTCC
	R	AGCCAAATCACCCGTCCTCTC
Bglap	F	AGCAGGAGGGCAATAAGGTAGTG
	R	CTCGTCACAAGCAGGGTTAAGC
Bglap2	F	AGCAGGAGGGCAATAAGGTAGTG
	R	CTCGTCACAAGCAGGGTTAAGC
Bglap3	F	CGCTCTGTCTCTCTGACCTCAC
	R	CGGAGTCTGTTCACTACCTTATTGC

Phex	F	TGATACCAGACTCTACCCACACTTG
	R	TCTTCCTCTCGGCACCTAATATCC
Mt1	F	CCCAACTGCTCCTGCTCCAC
	R	GCAGCCCACGGGACAGC
Mt2	F	GCTCCTGTGCCTCCGATGG
	R	CGGAAGCCTCTTTGCAGATGC

Table S2. Primer sequence of BMMs.

Genes		Primers（5′-3′）
Gapdh	F	TGACCACAGTCCATGCCATC
	R	GACGGACACATTGGGGGTAG
Trap	F	TGACCACAGTCCATGCCATC
	R	GACGGACACATTGGGGGTAG
Ctsk	F	TCAGGAACCACGGAATCCTC
	R	ACATTCAAGCGGATGCGTCT
Nfatc1	F	CCCAACTGCTCCTGCTCCAC
	R	GCAGCCCACGGGACAGC
Mmp9	F	CTGGACAGCCAGACACTAAAG
	R	CTCGCGGCAAGTCTTCAGAG

Table S3. Primer sequence of neutrophils.

Genes		Primers（5′-3′）
Gapdh	F	TGACCACAGTCCATGCCATC
	R	GACGGACACATTGGGGGTAG
IL-1β	F	TCGCAGCAGCACATCAACAAGAG
	R	AGGTCCACGGGAAAGACACAGG
IL-6	F	CTTCTTGGGACTGATGCTGGTGAC
	R	AGGTCTGTTGGGAGTGGTATCCTC
TNF-α	F	GCGACGTGGAACTGGCAGAAG
	R	GCCACAAGCAGGAATGAGAAGAGG
Cxcl10	F	TGCCTCATCCTGCTGGGTCTG
	R	TCCCTATGGCCCTCATTCTCACTG
S100a8	F	AAATCACCATGCCCTCTACAAG
	R	CCCACTTTTATCACCATCGCAA
S100a9	F	ATACTCTAGGAAGGAAGGACACC
	R	TCCATGATGTCATTTATGAGGGC
Pad4	F	GCTGGATGCCTTTGGGAACCTG
	R	CGCTGCTGGAGTAACCGCTATTC
NE	F	GTGCCGCCGTCGTGTGAAC
	R	CCAAGGGTCCGCCAGAGTCC
